# Triterpenoid profiling and functional characterization of the initial genes involved in isoprenoid biosynthesis in neem (*Azadirachta indica*)

**DOI:** 10.1186/s12870-015-0593-3

**Published:** 2015-09-03

**Authors:** Avinash Pandreka, Devdutta S. Dandekar, Saikat Haldar, Vairagkar Uttara, Shinde G. Vijayshree, Fayaj A. Mulani, Thiagarayaselvam Aarthy, Hirekodathakallu V. Thulasiram

**Affiliations:** Chemical Biology Unit, Division of Organic Chemistry, CSIR-National Chemical Laboratory, Dr. Homi Bhabha Road, Pune, 411008 India; CSIR-Institute of Genomics and Integrative Biology, Mall Road, New Delhi, 110007 India

**Keywords:** *Azadirachta indica*, Triterpenoids, Quantitative profiling, Transcriptome

## Abstract

**Background:**

Neem tree (*Azadirachta indica*) is one of the richest sources of skeletally diverse triterpenoids and they are well-known for their broad-spectrum pharmacological and insecticidal properties. However, the abundance of Neem triterpenoids varies among the tissues. Here, we delineate quantitative profiling of fifteen major triterpenoids across various tissues including developmental stages of kernel and pericarp, flower, leaf, stem and bark using UPLC-ESI(+)-HRMS based profiling. Transcriptome analysis was used to identify the initial genes involved in isoprenoid biosynthesis. Based on transcriptome analysis, two short-chain prenyltransferases and squalene synthase (AiSQS) were cloned and functionally characterized.

**Results:**

Quantitative profiling revealed differential abundance of both total and individual triterpenoid content across various tissues. RNA from tissues with high triterpenoid content (fruit, flower and leaf) were pooled to generate 79.08 million paired-end reads using Illumina GA ΙΙ platform. 41,140 transcripts were generated by *d e novo* assembly. Transcriptome annotation led to the identification of the putative genes involved in isoprenoid biosynthesis. Two short-chain prenyltransferases, geranyl diphosphate synthase (AiGDS) and farnesyl diphosphate synthase (AiFDS) and squalene synthase (AiSQS) were cloned and functionally characterized using transcriptome data. RT-PCR studies indicated five-fold and ten-fold higher relative expression level of AiSQS in fruits as compared to leaves and flowers, respectively.

**Conclusions:**

Triterpenoid profiling indicated that there is tissue specific variation in their abundance. The mature seed kernel and initial stages of pericarp were found to contain the highest amount of limonoids. Furthermore, a wide diversity of triterpenoids, especially C-seco triterpenoids were observed in kernel as compared to the other tissues. Pericarp, flower and leaf contained mainly ring-intact triterpenoids. The initial genes such as AiGDS, AiFDS and AiSQS involved in the isoprenoids biosynthesis have been functionally characterized. The expression levels of AiFDS and AiSQS were found to be in correlation with the total triterpenoid content in individual tissues.

**Electronic supplementary material:**

The online version of this article (doi:10.1186/s12870-015-0593-3) contains supplementary material, which is available to authorized users.

## Background

**Fig. 1:**
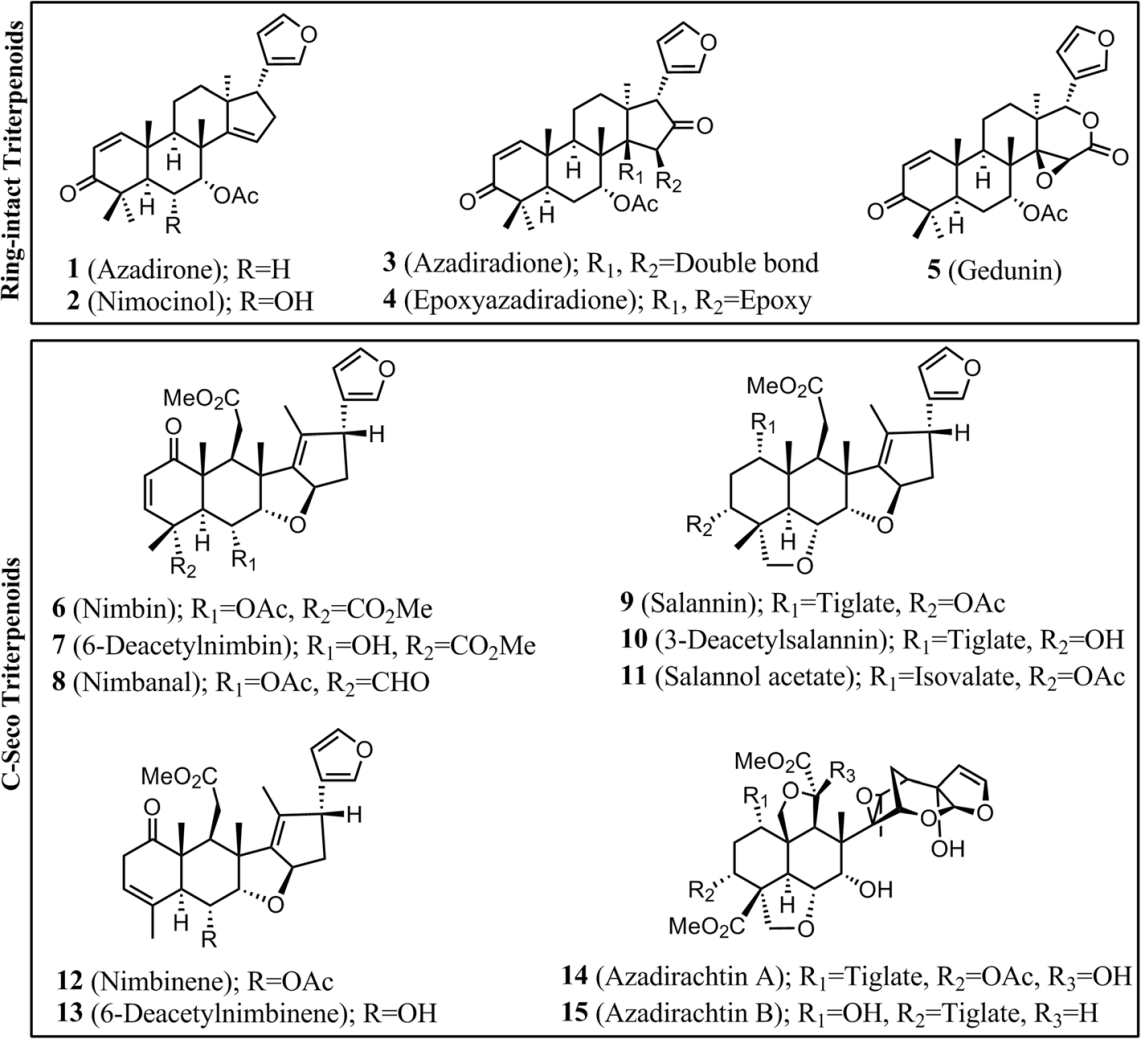
Skeletal diversity of Neem triterpenoids. Basic triterpenoids have azadirone, azadiradione, and gedunin type of skeletons. C- Seco triterpenoids have nimbin, salannin and azadirachtin type of skeletons

**Fig. 2:**
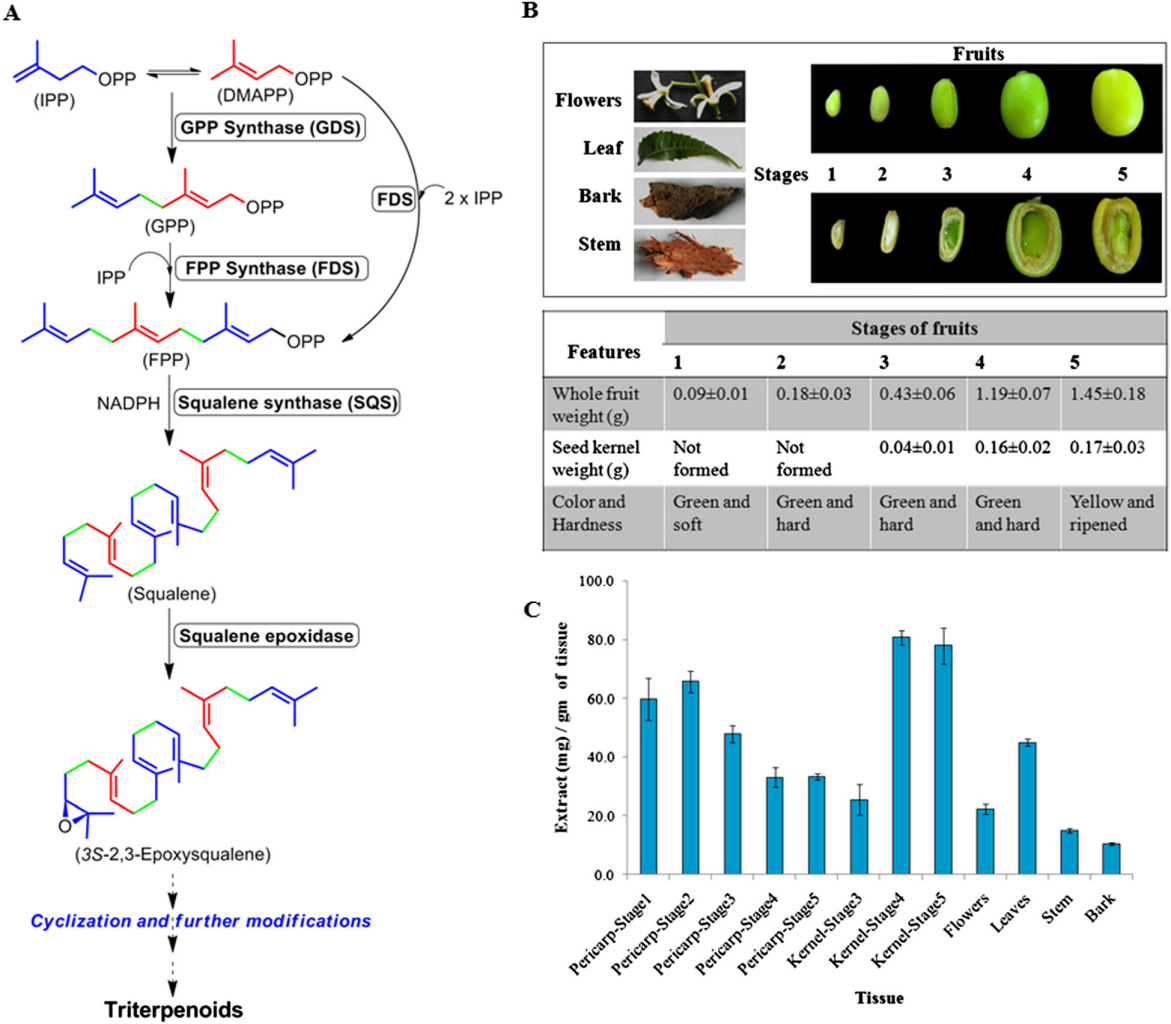
Predicted triterpenoid biosynthetic pathway, various Neem tissues and their total triterpenoids content in different tissues; (**a**) Initial genes involved in triterpenoid biosynthesis. **b** Different tissues of Neem and physical characteristics of Neem fruits from various stages. **c** Amount of triterpenoid extracts obtained from various tissues of Neem

Neem tree is one of the richest reserves of secondary metabolites, mainly tetranortriterpenoids (limonoids), which are known to be responsible for insecticidal and wide pharmaceutical activities [1, 2]. Various parts of this evergreen tree have been used as traditional medicine in day-to-day household remedies from ancient time. In addition to its therapeutic potential, Neem is being widely used in eco-friendly commercial pesticides and agrochemicals [3–5]. Over 150 structurally complex, highly oxygenated and skeletally diverse tetranortriterpenoids [2] have been isolated and characterized from different parts of the tree. Depending on the skeletal modifications, they can be categorized into two groups; ring-intact (basic) triterpenoids and C-seco triterpenoids [2, 6]. Ring-intact triterpenoids encompass 4,4,8-trimethyl-17-furanylsteroidal skeleton such as azadirone, azadiradione, and gedunin (**1**-**5**) type of structures (Fig. 1). C-seco triterpenoids are generated by the opening and further rearrangements of C-ring thus producing nimbin, salannin and azadirachtin (**6**-**15**) type of skeletons (Fig. 1). Although the biosynthetic pathway leading to the formation of triterpenoids (Fig. 2a) in Neem plant has been predicted [1, 7] genes involved in triterpenoid biosynthesis have not been characterized till date [8].

**Fig. 3:**
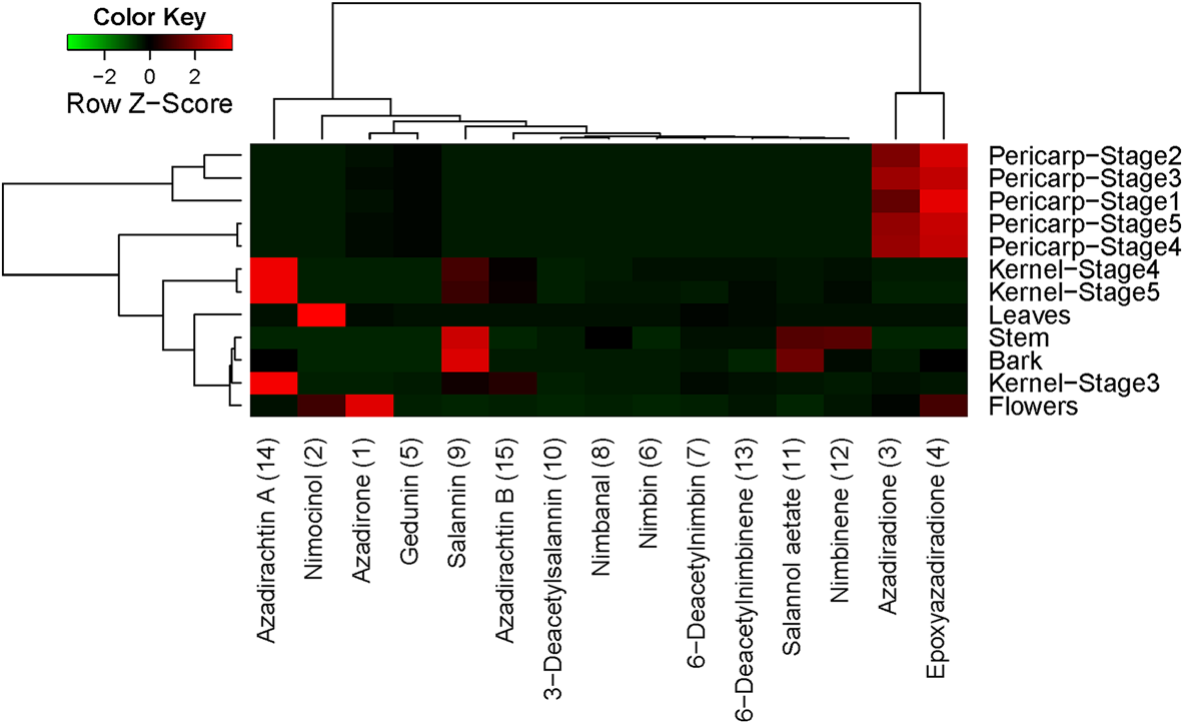
Quantitative abundance of major triterpenoids in different tissues of Neem. Basic and C-seco triterpenoids are highly abundant in Pericarp and Kernel respectively as compared to other tissues

Secondary metabolites are the final outcome of omics cascade and their distribution pattern is typical characteristic of every life in nature, which can be considered as an intrinsic signature of that species. Targeted metabolomics is all about identification and quantification of known metabolites and their time and space resolved distribution in a specific biological system [9–13]. Hyphenated mass spectrometry is a powerful and most utilized analytical technique in metabolomics due to its high sensitivity, accuracy, resolution, low sample requirement and ability to monitor broad range of metabolites [9, 12–14]. Triterpenoids in Neem are diverse in skeletal architecture, huge in count and their abundance is highly tissue-specific [1, 2]. Except few discrete studies [15, 16], there are no systematic investigations on the tissue- and stage-specific quantitative variation of Neem triterpenoids. It will be of great importance to investigate the targeted metabolic profiling of major triterpenoids in Neem plant, which may enlighten the differential tissue specific abundance of skeletally diverse triterpenoids. Further, correlation of metabolic profiling with transcriptome helps in analysis and identification of genes involved in Neem triterpenoid biosynthesis.

Terpenoid biosynthesis starts with basic building blocks such as Isopentenyl diphosphate (IPP) and dimethylallyl diphosphate (DMAPP) which are in turn synthesized through the mevalonate (MVA) or methylerythritol phosphate (MEP) pathways [17, 18]. Allylic diphosphate, DMAPP undergoes condensation with one or more IPP in head-to-tail fashion to produce linear diphosphates such as geranyl diphosphate (C_10_, GPP), farnesyl diphosphate (C_15_, FPP) and geranylgeranyl diphosphate (C_20_, GGPP) catalyzed by short-chain prenyltransferases such as geranyl diphosphate synthase (GDS), farnesyl diphosphate synthase (FDS) and geranylgeranyl diphosphate synthase (GGDS), respectively [19–21]. Two molecules of FPP undergo 1-1*'* head to head condensation to form squalene via NADPH dependent reduction of presqualene diphosphate intermediate catalyzed by squalene synthase (SQS) [22]. Thus squalene is the first committed precursor for the biosynthesis of triterpenoids [23]. This molecule is also well known to serve as a precursor for the primary metabolites such as steroids required for cell growth and division. Squalene thus acts as an important intermediate governing the balance between primary and secondary metabolism. Squalene undergoes further oxidation to form 2,3-epoxysqualene mediated by squalene epoxidase, followed by cyclization catalyzed by triterpene cyclases to form basic triterpene skeletons [24, 25]. Structural diversity of triterpenoids arises from the modifications of functional groups and rearrangements on the parental backbone of these triterpenes (Fig. 1) [26].

Short-chain prenyltransferases, such as FDS and SQS are shown to play key regulatory role in triterpenoid and phytosterol biosynthesis. To show some instances, when hairy root culture of *Panax ginseng* was treated with methyl jasmonate (MJ) to enhance the production of triterpenoids, FDS was up-regulated [27]. Over expression of mevalonate-5-pyrophosphate decarboxylase and FDS in *Panax ginseng* hairy root culture resulted in increased accumulation of phytosterols and triterepenes [28]. In *Centella asiatica*, overexpression of *Panax ginseng* FDS resulted in overexpression of dammarenediol synthase and cycloartenol synthase and when induced with MJ, enhanced production of triterpenes was observed [29]. Similarly, overexpression of SQS in *Panax ginseng*, *Eleutherococcus senticosus*, *Withania coagulans* and *Arabidopsis thaliana* showed increased production of phytosterols and triterpenoids [30–33]. Therefore, identification and functional characterization of short-chain prenyltransferases and SQS will assist in understanding of triterpenoid biosynthesis.

In this study, fifteen major triterpenoids were quantified in six different Neem tissues including kernel, pericarp, flower, leaf, stem and bark using UPLC-ESI(+)-HRMS based targeted profiling. Tissue specific profiling of triterpenoids delineated the variation in the abundance of triterpenoids across various tissues. This information was further utilized for the selection of tissues for transcriptome analysis followed by identification of initial genes involved in isoprenoid biosynthesis. Amongst the predicted genes from this pathway, here we report, molecular cloning and functional characterization of full-length geranyl diphosphate synthase (AiGDS), farnesyl diphosphate synthase (AiFDS) and squalene synthase (AiSQS) from Neem. Furthermore, using real-time PCR analysis, we showed that the expression level of one of the important genes in the pathway, AiSQS correlates with the triterpenoid content in respective tissues (fruit, leaf and flower).

## Results and discussion

### Tissue specific quantitative profiling of triterpenoids

The levels of individual fifteen triterpenoids (Fig. 1) were determined in different tissues of Neem including flowers, leaves, stem, bark, five developmental stages of pericarp and three stages of kernel (Additional file 1: Figure S5). The developmental stages of the fruits were classified on the basis of kernel formation, weight, hardness and colour (Fig. 2b). The crude mixture of triterpenoids was extracted from fresh tissues of Neem using solvent partition technique and were analyzed by UPLC-ESI(+)-HRMS in a gradient solvent program of methanol-water. Amount of crude extract obtained was directly correlated with the triterpenoid content of the corresponding tissue (Fig. 2c). Quantification of the crude extract revealed that kernel of stages 4 and 5 contained the highest amounts of triterpenoids (~80 mg/g of the tissue) followed by pericarp of stages 1, 2 and 3 (~48-66 mg/g). Pericarps of stages 4, 5 and kernel of stage 3 were found to possess comparatively lower amount of triterpenoids in the range of ~25-35 mg/g. Flowers and leaves have been shown to contain 22 and 45 mg/g of triterpenoids (including chlorophyll and other pigments), while stem and bark furnished 15 and 10 mg/g of the tissue respectively.

Standard graphs were prepared for each of the fifteen isolated triterpenoids within the concentration range of 0.04 to 0.003 mg/mL with injection volume 5 μL in UPLC-ESI(+)-HRMS (Additional file 1: Figure S4). They were further utilized for the quantification of individual molecules in the extracts of different tissues of Neem by correlating with the area under respective peaks of extracted ion chromatograms (Additional file 1: Figures S2 and S3). The quantitative level of individual fifteen triterpenoids across various tissues of Neem has been represented in Additional file 1: Figures S3 and S6. Among the fifteen triterpenoids under investigation, azadirachtin A (**14**), a well-studied Neem triterpenoid was found to be highly abundant in seed kernels, especially in the stages 4 and 5 (~3.6 mg/g of the tissue). Pericarp, flowers and leaves showed 100-500 fold lower levels (~0.004-0.04 mg/g) of azadirachtin A as compared to the kernel, whereas bark and stem contained negligible quantities (≤0.005 mg/g, 1000 fold lesser than seed kernel). Similar distribution was observed with the levels of azadirachtin B (**15**). Highest level of azadirachtin B was observed in kernel of stages 4 and 5 (0.5-0.6 mg/g), whereas pericarp and flowers showed 100-150 fold lesser amounts in comparison. Stem and bark were found to possess negligible levels (<0.005 mg/g, 1000 fold lesser than seed kernel) of azadirachtin B. Salannin (**9**) showed highest levels in kernel of stages 4 and 5 (1.2-1.4 mg/g). Salannin content was 4 fold less (~0.3 mg/g of the tissue) in stem as compared to that in kernel. Salannin content in bark was ~0.04 mg/g which was 35 fold lesser in comparison to seed kernel. Flowers, leaves and pericarp showed negligible levels of salannin (≤0.02 mg/g). Highest percentage of 3-deacetylsalannin (**10**) was observed in kernel of stages 4, 5 and stem with 0.01 mg/g of the tissue. Other tissues showed traceable amounts of 3-deacetylsalannin. Nimbin (**6**) was mainly present in kernels in the range of 0.1-0.2 mg/g and in negligible quantities in other tissues. 6-Deacetylnimbin (**7**) was found to be present in kernel of stages 4, 5 and leaves (0.08-0.23 mg/g). Nimbinene (**12**) and 6-deacetylnimbinene (**13**), two pentanortriterpenoids exhibited similar pattern of distribution across different tissues. Highest level was observed in seed kernels of stages 4, 5 and stem within the range of 0.15-0.25 mg/g. Flowers and leaves showed minor quantity (0.02-0.06 mg/g), whereas bark and pericarps exhibited negligible level. Nimbanal (**8**) was present in higher level in kernel of stages 4, 5 and stem (0.05-0.10 mg/g) and traceable levels were observed in other parts. Salannol acetate (**11**) was found to be abundant in seed kernels and stem with ~0.15 mg/g and in other tissues in minor amounts. Ring-intact triterpenoids (basic limonoids) such as azadirone, azadiradione, epoxyazadiradione and gedunin were found to be present at higher levels in pericarps. Azadiradione (**3**) showed highest level (3.0-8.0 mg/g) in all five developmental stages of pericarps especially in the stages 2 and 3 (7.0-8.0 mg/g), during which the seed kernel formation is about to start. These levels were about 100-200 fold higher than that in seed kernels (kernel stage 4 and 5) and flowers (0.01-0.05 mg/g). Other tissues contained negligible amounts of it (<0.001 mg/g). Similarly, epoxyazadiradione (**4**) showed 400-500 folds higher level in pericarps (9.0-12.0 mg/g; in stages 2 and 3) in comparison to that in the seed kernels (0.01-0.04 mg/g) and 50 folds higher than in flowers (~0.20 mg/g). Azadirone (**1**) was also found to be most abundant in all the developmental stages of pericarps (0.3-0.7 mg/g) especially in the stages 2 and 3 (0.6-0.7 mg/g) and flowers (0.5 mg/g). Leaves showed very less quantity (~0.08 mg/g) of **1** whereas other tissues contained traceable amounts (<0.001 mg/g). Gedunin (**5**), a potent anti-carcinogenic triterpenoid was abundantly present in pericarps, especially in the stages 2 and 3 (~1.0 mg/g). Negligible amount of **5** was present in other tissues (<0.002 mg/g). Nimocinol (**2**), 6α-hydroxy derivative of azadirone was observed to be abundant in leaves (2.9 mg/g), 15 fold higher than flowers (0.18 mg/g) and 50-150 times higher than pericarps (0.02-0.08 mg/g). Other tissues such as kernel, bark and stem showed very less amount of nimocinol (<0.001 mg/g).

Metabolic profiling data (Fig. 3 and Additional file 1: Figure S6) depicted the kernel to be rich in quantity and diversity of triterpenoids especially C-seco triterpenoids of azadirachtin (**14**, **15**), salannin (**9**, **11**), nimbin (**6**, **7**, **8**) and nimbinene (**12**, **13**) skeletons. However, pericarps were found to be rich in triterpenoids mainly consisting of ring-intact (basic) structures such as azadirone (**1**), azadiradione (**3**), epoxyazadiradione (**4**) and gedunin (**5**). Flowers and leaves showed relatively lower levels of triterpenoids and mostly of ring-intact skeletons (**1**, **2**, **3**, **4**). Stem and bark contained very low levels of triterpenoids; majorly C-seco metabolites of salannin (**9**, **11**) and nimbinene (**12**, **13**) type. In essence, profiling data revealed C-seco triterpenoids (**6-10**) to be the major constituents of triterpenoid pool from seed kernel, stem and bark whereas ring-intact skeletons (**1-5**) were observed to be major metabolites of the triterpenoid content obtained from pericarp, flower and leaf.

### Transcriptome analysis

For extensive coverage, RNA isolated from triterpenoid rich tissues such as fruit stage 4, leaves and flowers were pooled and used for transcriptome sequencing. A total of 79,079,412 (79.08 million) paired-end reads each of 72 bp length were generated by Illumina GA II platform. 71,537,895 (90.46 %) high quality reads were obtained with more than 20 phred score and reads of low quality were trimmed and used for further analysis. Total 27,390 contigs were generated using Velvet with a hash length of 41. These contigs were given as input for Oases to generate 41,140 transcripts. The average length of transcripts obtained was 1331 bp and the N50 length was 1953 bp (Table 1).

**Table 1:** Summary of transcriptome sequencing and assembly

Total number of reads	79079412
Total number of HQ reads	71537895
Hash length	41
Contigs generated	27390
Average contig length	897.431
N50 length of contigs	1479
Transcripts generated	41140
N50 length of transcripts	1953
Number of reads assembled	68871778

**Fig. 4:**
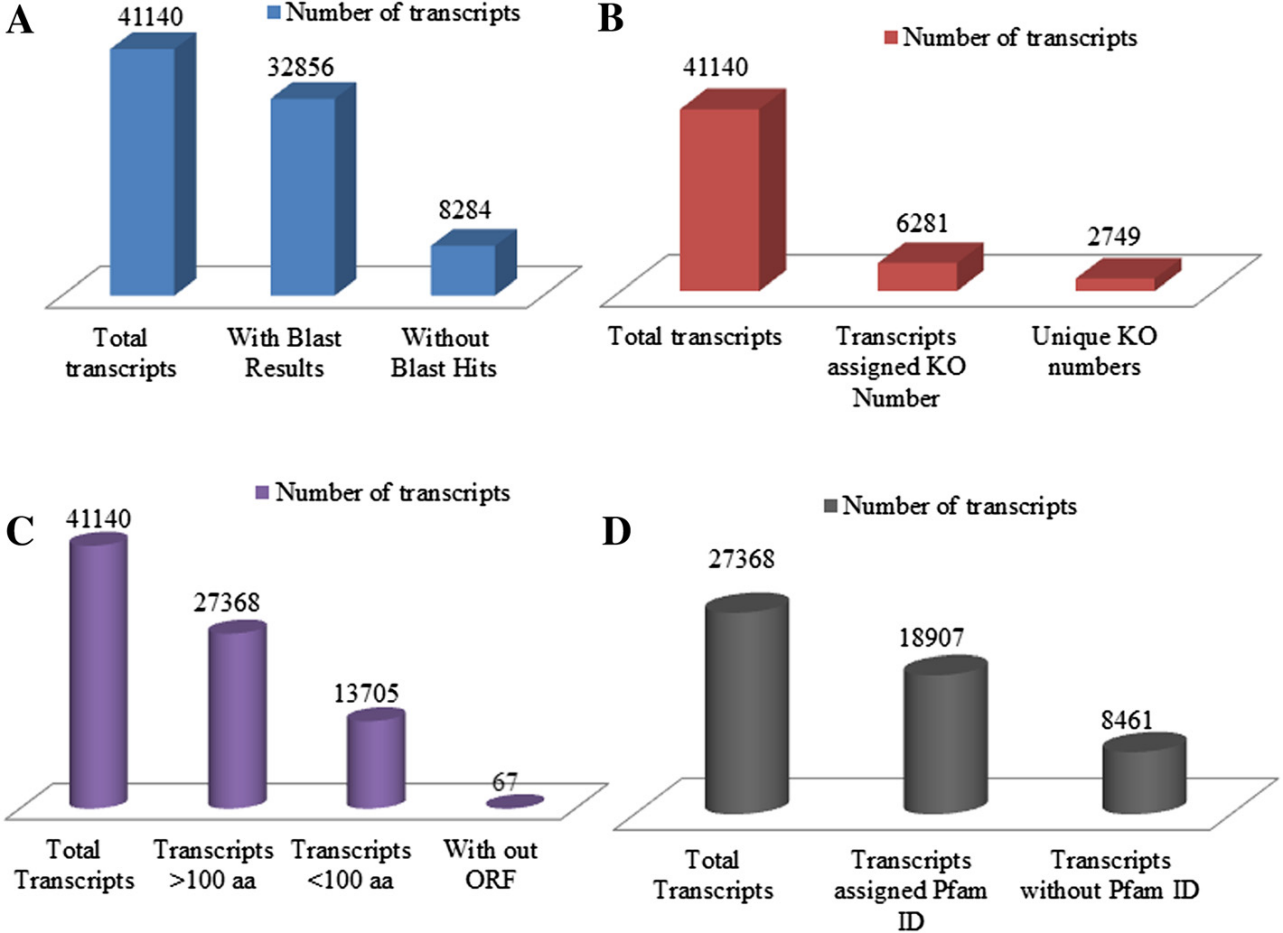
Functional annotation of transcriptome; (**a**) Based on Blastx analysis 80 % (32,856) transcripts had homologous proteins in NCBI nr database. **b** Based on KAAS analysis only 15.2 % (6281) transcripts were assigned 2749 KO numbers. **c** Based on virtual ribosome analysis 66.5 % (27,368) transcripts had ORF region length more than 100 amino acids and 0.001 % (67) Transcripts did not show ORF region. **d** Based on Pfam analysis 69.1 % (18,907) transcripts were assigned Pfam IDs

All the transcripts were submitted to Blastx against non-redundant database available at NCBI with an E-value cutoff of 10^-5^, where, a total of 32,856 (79.8 %) transcripts were annotated (Fig. 4a). Pathway annotation was carried out by KAAS (KEGG Automatic Annotation Server) with *Arabidopsis thaliana* (thale cress) and *Oryza sativa japonica* (Japanese rice) as the reference database. Out of the 41,140 transcripts only 6281 transcripts were assigned 2749 unique KO numbers, which covered 223 pathways (Fig. 4b). Virtual ribosome, a web based server, was used for finding the Open Reading Frame (ORF) of transcripts. 27,368 transcripts had an ORF with length more than 99 amino acids and 67 transcripts without any ORF (Fig. 4c). The peptide sequences of transcripts with length more than 99 amino acids were submitted to Pfam analysis. 18,807 transcripts were assigned different Pfam IDs. A total of 3467 different Pfam IDs were assigned to the transcripts (Fig. 4d). Based on transcriptome annotation, all the genes involved in triterpenoid back-bone biosynthesis from isoprene units (MVA pathway and MEP pathway) to triterpene cyclase were found (Additional file 1: Table S1). A total of 134 transcripts predicted as cytochrome P450 monooxygenases and two transcripts as cytochrome P450 reductases were identified. Based on BLAST results, with reference to *Arabidopsis thaliana* cytochrome P450, Neem CYP450s were classified into 39 families and 78 subfamilies, out of which most of the CYP450 belonged to CYP71 family. Seven transcripts were related to plant steroid biosynthesis and six transcripts related to triterpenoid biosynthesis were predicted (Additional file 1: Table S1). Recently, Neem draft genome and transcriptome of fruit, stem, leaf and flower [34], and suppression subtractive hybridization of transcripts between fruit mesocarp and endocarp [35] have been reported. However, there are no reports regarding functional characterization of the genes involved in Neem triterpenoid biosynthesis. To further explore this pathway, two short-chain prenyltranferases and squalene synthase were selected for functional characterization based on the transcriptome data.

### Heterologous expression and functional characterization of short-chain prenyltransferases (AiGDS and AiFDS)

Short-chain prenyltransferases function at the branching point of terpenoid metabolism and play regulatory role in the distribution of isoprene units into various terpenoids biosynthesis. In total, 12 short-chain prenyltranferases from Neem transcriptome were identified (Additional file 1: Table S1). Based on functional annotation studies, two geranyl diphosphate synthases (GDS), nine putative geranylgeranyl diphosphate synthases (GGDS) and one farnesyl diphosphate synthase (FDS) were identified. Sequence analysis using BLAST indicated that Neem_transcript_10912 was a homomeric GDS and Neem_transcript_10001 could be the smaller subunit of heteromeric GDS. TargetP analysis showed that both of these genes are localized in the mitochondria (Additional file 1: Table S5). For further study, Neem_transcript_10912 (AiGDS) and Neem_transcript_25722 (AiFDS) were selected for cloning and functional characterization.

**Fig. 5:**
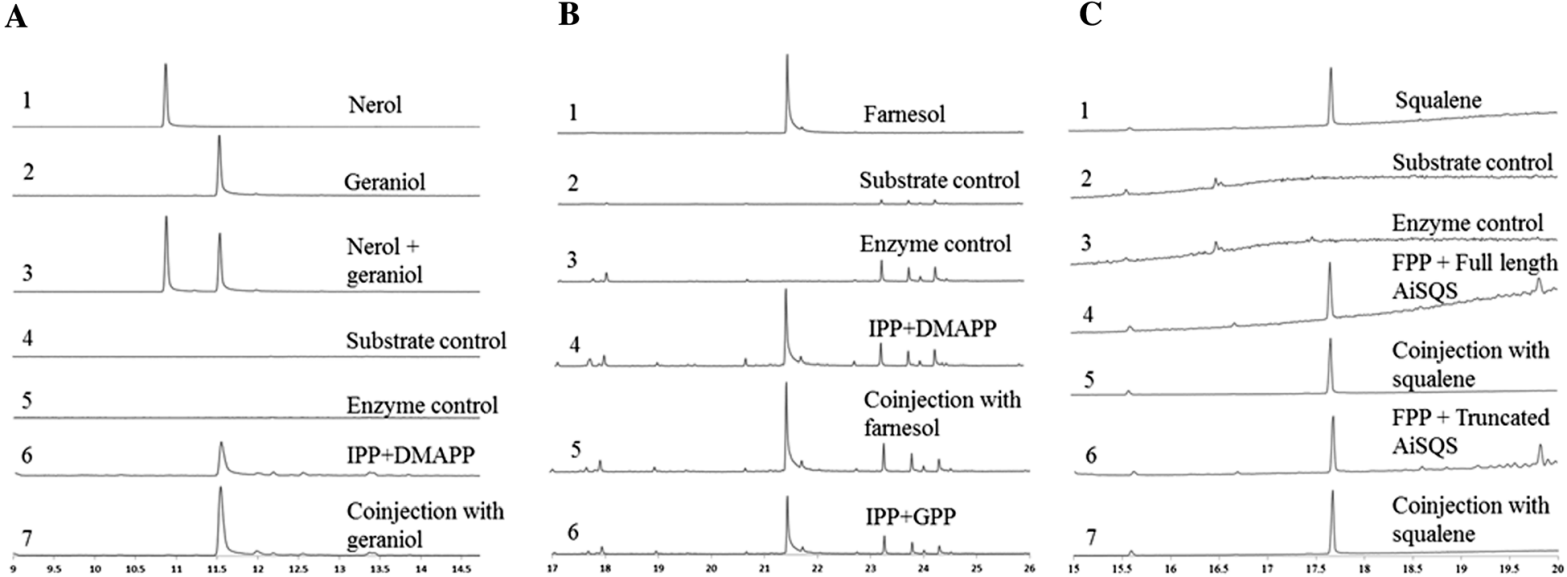
Total ion chromatograms (TICs) of AiGDS, AiFDS and AiSQS assays and relative expression level of AiSQS; (**a**) TICs of AiGDS assays; (1) Standard Nerol, (2) Standard geraniol, (3) Co-injection of standard nerol and geraniol, (4) Substrate control, (5) Enzyme control, (6) AiGDS enzyme assay with IPP and DMAPP as substrates, (7) Co-injection of standard geraniol with AiGDS enzyme assay extract. **b** TICs of AiFDS assays; (1) Standard (*E*,*E*)-farnesol, (2) IPP and DMAPP substrate control, (3) Enzyme control, (4) AiFDS enzyme assay with IPP and DMAPP as substrates, (5) Co-injection of standard (*E*,*E*)-farnesol and extract of AiFDS enzyme assay with IPP and DMAPP as substrates, (6) Extract of AiFDS enzyme assay with GPP and IPP as substrates. **c** TICs of AiSQS assays; (1) Standard squalene, (2) Substrate control, (3) Enzyme control, (4) Extract of full length AiSQS enzyme assay with FPP as substrate and NADPH as co-factor, (5) Co-injection of standard squalene and AiSQS enzyme assay extract, (6) Extract of truncated AiSQS enzyme assay with FPP as substrate and NADPH as co-factor and (7) Co-injection of standard squalene and truncated AiSQS enzyme assay extract

The ORF of AiGDS [GenBank: KM108315] was 1263 bp, which coded for a protein of 420 amino acids with theoretical molecular weight and calculated pI as 46.1 kDa and 6.33, respectively. AiGDS had maximum identity with several plant characterized homomeric GDSs such as 90 % identity to homomeric GDS from *Citrus sinensis* [GenBank: CAC16851] [36], 86 % identity to GDS from *Mangifera indica* [GenBank: AFJ52721] [37] and 76 % identity to GDS from *Catharanthus roseus* [GenBank: AGL91647] [38]. The percentage identity matrix of AiGDS with other plant homomeric GDS and heteromeric GDS larger subunits indicated that AiGDS possesses 71 % to 89 % identity with homomeric GDS (Additional file 1: Table S2). The multiple sequence alignment of AiGDS consisted of two aspartate rich motifs DDX_(2-4)_D and DDXXD which are highly conserved motifs in prenyltransferases and involved in substrate and metal ion binding (Additional file 1: Figure S7). CxxxC motifs were not observed in AiGDS, which play a key role in the interaction of heteromeric GDS [39]. The ORF of AiGDS was cloned into pET32a expression vector having an N-terminal thioredoxin domain and subsequently expressed in BL21 (DE3) cells. However recombinant AiGDS protein was found in inclusion bodies. To enhance solubility, AiGDS cloned construct was transformed into Lemo 21 (DE3) cells [40] and expression was carried out. Recombinant AiGDS protein remained solely in the insoluble portion in the pellet. Eventually we were able to obtain soluble active AiGDS by re-suspending the pellets in lysis buffer, then drop-wise addition of 0.1 M NaOH until pH 11.0 with constant swirling on ice till the solution became clear. The pH was then reduced to 7.0 using 0.1 M HCl under similar conditions [41]. The resulting solution was centrifuged at 10,000 × *g* and subjected to SDS-PAGE analyses (Additional file 1: Figure S11A). The AiGDS was found to be in soluble form in the supernatant, which was subjected to purification by Ni-NTA affinity chromatography. The recombinant protein was over 94 % pure as analysed by SDS-PAGE (Additional file 1: Figure S11A). Purified recombinant AiGDS was incubated with equimolar concentration of IPP and DMAPP followed by treatment with alkaline phosphatase to hydrolyze the diphosphate esters to their corresponding alcohols. The extracted assay mixture was analyzed by GC-MS and the products formed were confirmed by comparing the retention time and coinjection studies with standard geraniol (Fig. 5a). GC-MS analyses of the extracts of alkaline phosphatase treated assay mixture of AiGDS with GPP/FPP and IPP indicated that AiGDS failed to synthesize chain elongation products FPP (C_15_) or GGPP (C_20_) suggesting that AiGDS can catalyse the chain elongation reaction to produce GPP (C_10_) as sole enzymatic product.

AiFDS [GenBank: KM10831] ORF of 1029 bp length was found to be encoding for a protein of 342 amino acids. The theoretical molecular weight and pI for this polypeptide were 39.5 kDa and 5.59 respectively. The sequence comparison of AiFDS exhibited 83 % identity with FDS from *Mangifera indica* [GenBank: AFJ52720] [37], 82 % identity with that from *Santalum album* [GenBank: AGV01244.1] and 81 % identity with FDS from *Catharanthus roseus* [GenBank: ADO95193.1] [42]. The multiple sequence alignment of AiFDS consisted of two aspartate rich motifs DDX_(2-4)_D and DDXXD (Additional file 1: Figure S8) which were highly conserved motifs in prenyltransferases. AiFDS was cloned into pET32a expression vector. The cloned construct was transformed into BL21 (DE3) cells and expressed. AiFDS was obtained as soluble form and purified by Ni-NTA affinity column chromatography. The recombinant protein was over 98 % pure as analyzed by SDS-PAGE (Additional file 1: Figure S11B). Buffers used for AiGDS and AiFDS protein purification are given in Addition file 1: Table S4. The purified short-chain prenyltransferase was incubated with DMAPP/GPP and IPP followed by treatment with alkaline phosphatase. GC-MS analyses of the assay extracts indicated the formation of FPP which was further confirmed by comparing the retention time, mass fragmentation pattern and coinjection studies with standard (*E,E*)-farnesol (Fig. 5b). Further GC-MS analysis of alkaline phosphatase treated assay mixture of AiFDS with FPP and IPP did not show formation of geranylgeraniol indicating that AiFDS catalyses the chain elongation reaction to produce FPP as the sole enzymatic product**.**

### Heterologous expression and functional characterization of squalene synthase (AiSQS)

An ORF of 1176 bp encoding a polypeptide of 396 amino acids was identified as AiSQS [GenBank: JQ327160]. The theoretical pI of protein was found to be 8.18 and molecular weight of 44 kDa. The amino acid sequence of AiSQS shared 86 % identity with squalene synthase from *Diospyros kaki* [GenBank: ACN69082], 85 % identity with *Camellia oleifera* [GenBank: AGB05603], 84 % identity with *Euphorbia tirucalli* [GenBank: BAH23428] and 84 % identity with that from *Glycyrrhiza glabra* [GenBank: BAA13084.1]. Eukaryotic SQSs have four conserved regions and are important for catalysis as indicated by biochemical characterization of site-directed mutants and crystal structure of human squalene synthase [43] (Additional file 1: Figure S9). The aspartate rich motifs found in region 1 and 3 are involved in binding of the diphosphate moiety of FPP via bridging Mg^2+^ ions. Careful analysis of AiSQS sequence with TMHMM program showed the presence of transmembrane motif YNTTMIIMLFIILAIIFAYLSAN at the C-terminus. Although transmembrane domain exhibits low level of sequence homology with other SQS enzymes, this domain is highly hydrophobic and consistent with the putative endoplasmic reticulum anchoring function.

Squalene synthase has been characterized previously from human [43], rodents [44, 45], plants [46–48], protozoa [49] and fungi [50]. All these SQS enzymes were obtained in soluble form by deletion of a putative C-terminal membrane-spanning motif [51]. In the present study we have cloned the full-length ORF of AiSQS, as well as a truncated AiSQS by deletion of 15 amino acids from N-terminal and 63 amino acids from the C-terminal end into pRSET-C and pET28c vectors respectively. The truncated AiSQS was transformed into BL21 (DE3) cells, expressed and purified by subjecting to Ni-NTA affinity column chromatography. Purified truncated AiSQS was analyzed by SDS-PAGE which showed a single band (>90 % purity) at ~35 kDa, consistent with the predicted molecular mass for the (His)_6_-tagged enzyme (Additional file 1: Figure S11D).

The full-length recombinant AiSQS protein was expressed in BL21 star (DE3) cells. Majority of the protein was found to be insoluble (Additional file 1: Figure S11C). Lee and Poulter observed that adding glycerol to the lysis and purification buffers helped in solubilization of the insoluble *T. elonatus* BP-1 SQS [52]. Induced cell pellets were disrupted in lysis buffer containing 50 % (v/v) glycerol and 1 % CHAPS. The glycerol concentration in cell lysate obtained was reduced to 20 % (v/v) by adding lysis buffer (without glycerol). This lysate was subjected to Ni-NTA affinity column chromatography. The purified full length AiSQS, when analyzed by SDS-PAGE, exhibited a single band (90 % purity) at approximately 44 kDa, consistent with the predicted molecular mass for the (His)_6_-tagged enzyme (Additional file 1: Figure S11C). Purified proteins were flash-frozen in liquid nitrogen and stored at -80 °C until further use. Buffers used for AiSQS full length and truncated protein purification are given in Addition file 1: Table S4.

GC-MS analyses of the assay extracts of full length and truncated AiSQS with FPP in the presence of NADPH indicated the formation of squalene. The formation of squalene was further confirmed by comparing the retention time, mass fragmentation pattern and co-injection studies with standard squalene (Fig. 5c). This confirms that AiSQS catalyzes the condensation of two molecules of farnesyl diphosphate (FPP) to form squalene through a NADPH-dependent rearrangement of C1′-2-3-linked triterpene intermediate, presqualene diphosphate [52].

### Real time PCR analysis

To determine the role of short-chain prenyl diphosphate synthases and squalene synthase in triterpenoid biosynthesis, real time PCR analysis of the Neem_transcript_10001 (smaller subunit of heteromeric geranyl diphosphate synthase), AiGDS, AiFDS, and AiSQS was carried out.

**Fig. 6:**
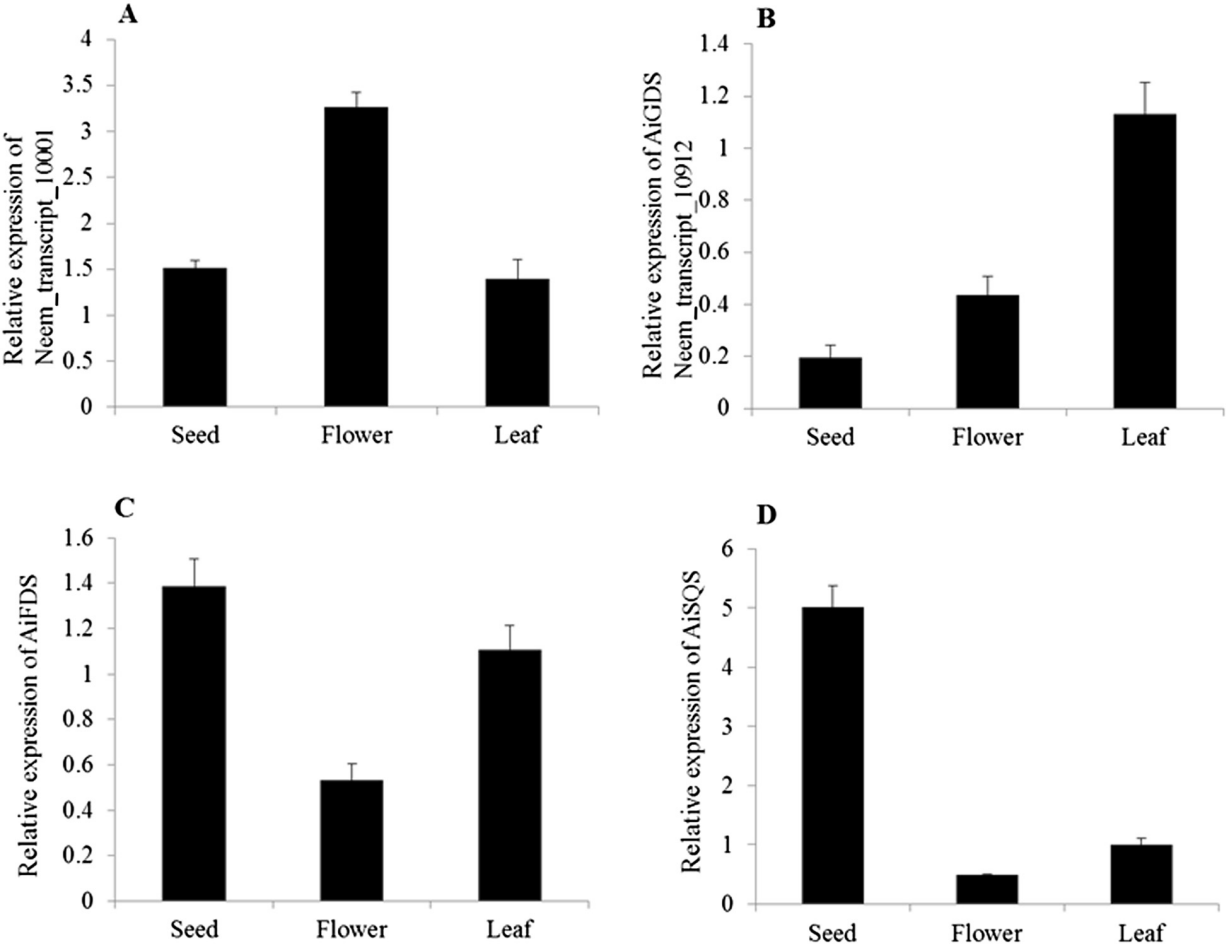
Real-time PCR analysis. **a** Neem_transcript_10001 showed very high expression in flower. **b** AiGDS was highly expressed in leaf. **c** AiFDS has higher expression level in seeds. **d** Relative expression levels of AiSQS was very high in seeds as compared to other tissues. Error bars represents standard error

AiSQS is the first committed enzyme involved in triterpene biosynthesis in Neem. Real time PCR was carried out for AiSQS from flowers, leaves and fruit and normalized with 18S rRNA expression level. Neem fruit showed fivefold higher expression level in comparison with the leaves and tenfold higher relative expression level than flowers (Fig. 6d). The results were in correlation with profiling of triterpenoids from different tissues. Neem fruits as a whole, not only showed structurally diverse triterpenoids but also showed very high levels of these metabolites. On the other hand, flowers and leaves exhibited lesser skeletal diversity and quantity of abundant triterpenoids. Squalene is the precursor of primary metabolites such as membrane sterols and steroid hormones required for cell division and growth. Also, it serves as precursor for triterpenoids found in Neem, which assign squalene, a crucial branch point between primary and secondary metabolism. Transgenic *Panax ginseng* overexpressing squalene synthase has previously shown to produce higher levels of triterpene and phytosterols than wild type strains which depict the key role of intracellular squalene flux between primary and secondary metabolism [31]. High expression levels of AiSQS in fruits indicated considerable amount of squalene flux might get diverted towards triterpenoids formation in Neem fruits.

AiFDS (Fig. 6c), compared to other tissues, showed very high expression levels in seeds. Similar expression patterns of AiFDS and AiSQS suggest that both these genes could be involved in triterpenoid biosynthesis. On the contrary, AiGDS (Fig. 6b) and Neem_transcript_10001 (Fig. 6a) showed very high expression in leaf and flower, respectively, compared to other tissues. These results indicate that AiGDS may not be involved in triterpene biosynthesis in Neem.

### Phylogenetic analysis

Neighbour joining phylogenetic tree was constructed based on the deduced amino acid sequences of AiGDS, AiFDS and AiSQS with corresponding enzymes from different organisms, which were retrieved from the NCBI GenBank database (Additional file 1: Figure S10). The degree of relatedness correlated well with the amino acid similarity among the plant proteins, which indicated AiGDS, AiFDS and AiSQS belonged to the clade of plant kingdom. These enzymes from Neem were classified into one cluster revealing their closest evolutionary relationships with the plant group.

## Conclusions

Due to immense significance of Neem as a wonder tree and known to synthesize biologically and commercially important triterpenoids having highly complex carbon skeleton with diverse functional groups, it is of great interest to study their biosynthetic pathway. Levels of total triterpenoid and fifteen major individual triterpenoids were quantified in various tissues of the Neem plant. Tissue specific variation in the abundance of triterpenoids has been observed. The mature seed kernel and pericarp of initial stages were found to contain the highest amount of triterpenoids. Furthermore, a wide diversity of triterpenoids, especially C-seco triterpenoids were observed in kernel as compared to the other tissues. Pericarp, flower and leaf contained mainly ring-intact triterpenoids. From transcriptome analysis, short-chain prenyl trasnferases, squalene synthase, squalene expoxidase, triterpene synthases and putative cytochrome P450 genes were predicted. The genes involved in the initial steps of isoprenoid biosynthesis, such as AiGDS, AiFDS and AiSQS were cloned and functionally characterized. Furthermore, AiFDS and AiSQS expression levels were found to be nicely correlating with the triterpenoids content of various tissues of Neem.

## Methods

### Materials and chemicals

Neem tissues for the profiling of triterpenoids were collected from Pune region, Maharashtra, India in the period March to May. Fifteen reference triterpenoids were isolated and characterized as reported earlier [53, 54, 6] and described briefly in Additional file 1. For extraction, HPLC grade solvents were purchased from Sigma (St. Louis, MO, USA). For UPLC-ESI(+)-MS experiments LC-MS grade solvents were procured from Avantor Performance Materials, JT Baker (PA, USA). SuperScript® III First-Strand Synthesis System (Invitrogen) was used for cDNA synthesis. For PCR amplification, AccuPrime™ (Invitrogen) polymerase was used. For Restriction digestion, NEW ENGLAND BioLabs®_inc_(NEB) restriction enzymes were used. Gel extraction of restricted product and vector were carried out by GenElute™ Gel Extraction Kit from Sigma. T_4_ DNA ligase from Invitrogen was used for ligation. TOP10 cells (Invitrogen) were used for cloning. Lemo21 (DE3) cells (NEB), BL21 (DE3) cells (NEB) and BL21 Star (DE3) cells (Invitrogen) were used as expression cells. Ni-NTA agarose (Invitrogen) was used for protein purification. Enzyme assay samples were analyzed on Agilent 7890A GC coupled with 5975C mass detector. Geraniol, nerol, (*E,E*)-farnesol, squalene standards were purchased from Sigma Aldrich. IPP, FPP, GPP, and DMAPP were synthesized as reported previously [55, 56].

### Extraction of total triterpenoids

Fresh Neem tissues (0.5 g) were extracted with methanol (10 mL × 3), by continuous stirring for 3 h. The pooled methanol layer after concentration under reduced pressure at 50 °C was partitioned between ethyl acetate (20 mL) and water (20 mL). The organic layer was separated, passed through anhydrous sodium sulphate and concentrated under similar conditions to obtain the crude triterpenoid extract. Extraction of individual tissues was performed in triplicates.

### UPLC-ESI(+)-HRMS profiling of triterpenoid extract

For triterpenoids profiling, UPLC-ESI(+)-HRMS runs were performed on Q Exactive Orbitrap associated with Accela 1250 pump (Thermo Scientific, MA, USA). Mixture of triterpenoids were dissolved in a known volume of methanol (concentration ~0.2 mg/mL), centrifuged to remove the suspended particles and injected (10 μL) in UPLC-ESI(+)-HRMS (Additional file 1: Figure S5). Samples were resolved through Acquity BEH C18 UPLC column (2.1 × 100 mm) of particle size 1.7 μM with a flow rate of 0.3 mL/min and gradient solvent program of 35 min (0.0 min, 40 % methanol/water; 5.0 min, 50.0 % methanol/water; 10.0 min, 60 % methanol/water; 25.0 min, 65 % methanol/water; 30.0 min, 90 % methanol/water; 32.0 min, 90 % methanol/water; 34.0 min, 40 % methanol/water; 35.0 min, 40 % methanol/water). 0.1 % LC-MS grade formic acid was also added to water (mobile phase). Profiling experiments were performed in ESI-positive ion mode using the tune method as follows: sheath gas (nitrogen) flow rate 45 units, auxiliary gas (nitrogen) flow rate 10 units, sweep gas (nitrogen) flow rate 2 units, spray voltage (|KV|) 3.60, spray current (μA) 3.70, capillary temperature 320 °C, s-lens RF level 50, heater temperature 350 °C. ESI(+)-HRMS data were recorded in full scan mode within the mass range *m/z* 100 to 1000. Profiling data were analyzed through Thermo Xcalibur software. Retention times (R_t_) and extracted ions for the individual studied triterpenoids have been listed in Table 2. UPLC-ESI(+)-HRMS chromatograms for individual standard triterpenoids and their corresponding ESI(+)-HRMS spectra have been provided in Additional file 1: Figures S2 and S3. R version 3.1.2 was used for generating heatmap.

**Table 2:** Retention times (R_t_), extracted ions and corresponding molecular fragments for the studied triterpenoids

Triterpenoid	R_t_ (min)	Extracted ion (*m/z*)	Fragment
Azadirachtin A (14)	7.4	703	[M + H-H_2_O]^+^
Azadirachtin B (15)	8.6	645	[M + H-H_2_O]^+^
Salannin (9)	21.3	597	[M + H]^+^
3-Deacetylsalannin (10)	18.5	555	[M + H]^+^
Nimbin (6)	18.0	541	[M + H]^+^
6-Deacetylnimbin (7)	14.7	499	[M + H]^+^
Nimbinene (12)	20.7	483	[M + H]^+^
6-Deacetylnimbinene (13)	16.5	441	[M + H]^+^
Nimbanal (8)	17.8	511	[M + H]^+^
Salannol acetate (11)	28.4	599	[M + H]^+^
Azadiradione (3)	16.3	451	[M + H]^+^
Epoxyazadiradione (4)	27.4	467	[M + H]^+^
Azadirone (1)	31.7	437	[M + H]^+^
Gedunin (5)	20.4	483	[M + H]^+^
Nimocinol (2)	29.9	453	[M + H]^+^

### Transcriptome analysis

Total RNA was isolated using Spectrum Plant total RNA isolation kit (Sigma-Aldrich). Equal quantity of RNA from each tissue was mixed. Transcriptome library was constructed using TruSeq RNA Sample Preparation Guide (Illumina). Quality of the prepared library was analyzed by running an aliquot on High Sensitivity Bioanalyzer Chip (Agilent). 79,079,412 paired end raw reads were generated with the length of 72 bp by Illumina GA ΙΙ analyzer. *De novo* assembly was carried out by Velvet (version- 1.1.05) with hash length 41 [57]. A total of 27,390 contigs were generated with average contig length of 897 and N50 value of 1479. These contigs were then submitted to Oases (version- 0.2.01) to generate a total of 41,140 transcripts [58]. Neem transcripts were submitted to Blastx against non-redundant database available at NCBI with E-value cutoff of 10^-5^. Pathway annotation was done by bidirectional best hit method of KAAS (KEGG Automatic Annotation Server. http://www.genome.jp/kegg/kaas) with *Arabidopsis thaliana* (thale cress) and *Oryza sativa japonica* (Japanese rice) as the reference database [59]. Virtual ribosome, (http://www.cbs.dtu.dk/services/VirtualRibosome/) a web based server, was used for deducing the ORFs of these transcripts [60]. The peptide sequences of transcripts with length more than 99 amino acids were submitted to batch search of Pfam (http://pfam.xfam.org/search#tabview=tab1) [61].

### Cloning and characterization of AiGDS, AiFDS and AiSQS

The Neem seed RNA was used for the synthesis of cDNA using SuperScript® III First-Strand Synthesis System (Invitrogen). Full length primers for AiGDS and AiFDS ORFs were designed using their transcripts as a template (Additional file 1: Table S3). Synthesized cDNA was used for PCR reaction using AccuPrime (Invitrogen). PCR products were cloned into pET32a expression vector using respective cloning sites. Full length and truncated primers for AiSQS were designed from Neem_transcript_33869 (Additional file 1: Table S3). PCR products were cloned into pCR Blunt vector. Further, the ORF was digested with EcoRI and the resulting fragment was ligated into pRSET-C vector for full length AiSQS and pET28c for truncated AiSQS. The expression of the recombinant plasmids containing AiGDS, AiFDS, truncated AiSQS were carried out in BL21 (DE3) cells except full length AiSQS, which was expressed in BL21 Star (DE3) cells.

Initially, AiGDS and full length AiSQS were found in inclusion bodies. Expression of AiGDS in Lemo 21 (DE3) cells did not show any improvement in the solubility. To obtain the soluble AiGDS protein, the pellet obtained after crude lysate centrifugation at 10,000 × *g* was resuspended in lysis buffer, pH was increased to 11.0 with 0.1 M NaOH and then reduced to 7.0 with 0.1 M HCl (pH adjustment was done on ice with continuous stirring). The resulting solution was centrifuged at 10,000 × *g* for 10 min at 4 °C [41]. The supernatant containing AiGDS protein was purified over Ni-NTA affinity chromatography by following user manual.

Purification of full length AiSQS was attempted under denaturing conditions in 50 mM Tris buffer containing 6 M guanidium hydrochloride as well as 8 M urea as denaturing agents. Refolding was attempted by stepwise slow removal of denaturants under dialysis. However, the protein obtained was not catalytically active. Purification under native conditions using buffer combinations of HEPES, TRIS, MOPS with non-ionic detergents like T ween 20, Triton X-100 also did not yield sufficient amount of soluble protein. A considerable amount of protein was found in soluble fractions using 50 % glycerol and 1 % CHAPS in Phosphate buffer. All the recombinant proteins were purified by Ni-NTA affinity chromatography. Buffers used for recombinant protein purifications were given in Addition file 1: Table S4. Protein estimation was performed by Bradford assay [62] and the protein purity was analyzed on SDS-PAGE (Additional file 1: Figure S11).

Enzyme assays for AiGDS and AiFDS were performed in HEPES buffer with DMAPP (100 μM)/GPP (100 μM) and IPP (100-200 μM) as substrates. 100 μM FPP was used as substrate for full length and truncated AiSQS with 1 mM NADPH as cofactor. The reaction mixtures were incubated at 30 °C for 2 h. AiSQS assay reaction was quenched by adding 1 M sodium hydroxide. For AiGDS and AiFDS assays, alkaline phosphatase (6 U) was added and further incubated at 37 °C for 1 h. Reaction mixtures were extracted thrice using n-hexane. Samples were concentrated with a stream of dry nitrogen and analysed by GC-MS on 30 m × 0.25 mm × 0.25 μm capillary columns (HP-5 and HP-5 MS, J & W Scientific). Functional characterization of AiFDS and AiGDS was carried out on GC-MS using the program: 70 °C for 1 min, 5 °C/min rise till 150 °C, 10 °C/min rise till 270 °C and hold for 5 min (Program 1). For the functional characterization of AiSQS, the program used was: initial temperature of 150 °C for 2 min followed by increase in temperature to 320 °C at the rate of 10 °C/min and hold at 320 °C for 11 min (Program 2). Product formation was confirmed by co-injection with authentic standards and comparing the mass fragmentation pattern and retention time (Fig. 5).

### RT-PCR analysis

Real time PCR was carried out using Super Script III platinum SYBR green one-step qRT-PCR kit (Invitrogen, USA). In brief, for AiSQS quantification, 100 ng of DNase treated total RNA was added with AiSQS primers and for 18S intrinsic control, 18S primers were used (Additional file 1: Table S3). cDNA synthesis and PCR were carried out in a single tube reaction. cDNA synthesis was performed at 50 °C for 5 min followed by denaturation at 95 °C for 5 min and subsequent 40 cycles of denaturation step at 95 °C for 3 s, combined annealing and extension step at 60 °C for 30 s per cycle.

Quantification of AiGDS, AiFDS and Neem_Transcript_10001, was performed as follows: Initial cDNA synthesis was performed at 50 °C for 20 min, followed by 95 °C for 5 min, 40 cycles of 95 °C for 10 s and 60 °C for 30 s. GAPDH primers were used as an endogenous control to normalize the expression levels between different tissues. Threshold (Ct) values were obtained and ΔCt was calculated as Ct target gene – Ct endogenous reference gene. Relative fold difference was calculated using 2^ΔCt^. Experiments were carried out using three biological replicates with five technical replicates each.

### Phylogenetic analysis

Reference protein sequences were obtained from GenBank database. Sequences were aligned using ClustalW using default parameters [63]. Neighbour joining tree was constructed with MEGA version 6.06 software [64]. Bootstrap analyses with 1000 replicates were also conducted in order to obtain confidence levels for the branches.

### Availability of supporting data

The Illumina RNA-seq data generated from pooled RNA from leaves, fruits and flowers of *Azadirachta indica* are available in the NCBI SRA (http://trace.ncbi.nlm.nih.gov/ Traces/sra) with accession SRR2145149.

## Additional file

Additional file 1: Methods 1. Isolation of Neem triterpenoids from seed kernel and pericarp. **Methods 2.** Characterization of purified Neem triterpenoids. **Figure S1.** TLC profile of crude extracts and purified triterpenoids (developed in 70 % ethyl acetate in n-hexane for twice). **Figure S2.** UPLC-ESI(+)-quadrupole/orbitrap-MS extracted ion chromatograms of the fifteen pure triterpenoids from Neem. Chromatograms have been arranged in the order of increasing retention time. **Figure S3.** ESI(+)-quadrupole/orbitrap-MS spectra of the fifteen pure triterpenoids from Neem. **Figure S4.** Standard graphs for the purified triterpenoids prepared in UPLC-ESI(+)-quadrupole/orbitrap-MS; concentration range 0.040-0.003 mg/mL, injection volume 5 μL. **Figure S5.** Representative UPLC-ESI(+)-quadrupole/orbitrap-MS chromatograms of various Neem tissue extracts (× denotes non-triterpenoids with molecular mass less than 350). **Figure S6.** Quantitative abundance of individual triterpenoids in different tissues of Neem. **Figure S7.** Multiple sequence alignment of *A. indica* geranyl diphosphate synthases (AiGDS). **Figure S8.** Multiple sequence alignment of *A. indica* farnesyl diphoshate synthase (AiFDS). **Figure S9.** Multiple sequence alignment of *A. indica* Squalene synthase (AiSQS); Amino acid sequence alignment of *C. annuum* (CaSQS, AAD20626), *N. tabacum* (NtSQS, AAB08578), *A. indica* (AiSQS, AFJ15526), *L. japonicas* (LjSQS, BAC56854), *G. max* (GmSQS, NP_001236365), *P. vulgaris* (PvSQS, AHA84150). The solid lines indicate four highly conserved regions 1, 2, 3 and 4 which are considered to be the catalytic sites of squalene synthases. **Figure S10.** Phylogenetic analysis of AiGDS, AiFDS and AiSQS. **Figure S11.** Purification of recombinant AiGDS, AiFDS and AiSQS. **Table S1.** Predicted genes for Triterpenoid back bone biosynthesis. **Table S2.** Present Identity Matrix of AiGDS with plant homomeric GDS and heteromeric GDS Larger subunits. **Table S3.** Primers and vectors used for cloning of AiGDS, AiFDS and AiSQS and RT-PCR primers of 18S rRNA, GAPDH, Neem_transcript_10001, AiGDS and AiSQS. **Table S4.** Buffers used for AiGDS, AiFDS and AiSQS protein purification. **Table S5.** TargetP analysis Neem_transcript_10912 (AiGDS) and Neem_Transcript_10001. (DOCX 3387 kb)

## Abbreviations

MVA: Mevalonate pathway
MEP: Methylerythritol phosphate pathway
GPP: Geranyl diphosphate
FPP: Farnesyl diphosphate
GGPP: Geranylgeranyl diphosphate
GDS: Geranyl diphosphate synthase
FDS: Farnesyl diphosphate synthase
GGDS: Geranylgeranyl diphosphate synthase
SQS: Squalene synthase
MJ: Methyl jasmonate
ORF: Open Reading Frame
