# Supplementary material for: Triterpenoid profiling and functional characterization of the initial genes involved in isoprenoid biosynthesis in neem (Azadirachta indica)
Source: BMC Plant Biol. 2015 Sep 3;15:214. doi: 10.1186/s12870-015-0593-3 (PMC4559364; doi:10.1186/s12870-015-0593-3)
Supplement: Additional file 1: Methods 1. — Isolation of Neem triterpenoids from seed kernel and pericarp. Methods 2. Characterization of purified Neem triterpenoids. Figure S1. TLC profile of crude extracts and purified triterpenoids (developed in 70 % ethyl acetate in n-hexane for twice). Figure S2. UPLC-ESI(+)-quadrupole/orbitrap-MS extracted ion chromatograms of the fifteen pure triterpenoids from Neem. Chromatograms have been arranged in the order of increasing retention time. Figure S3. ESI(+)-quadrupole/orbitrap-MS spectra of the fifteen pure triterpenoids from Neem. Figure S4. Standard graphs for the purified triterpenoids prepared in UPLC-ESI(+)-quadrupole/orbitrap-MS; concentration range 0.040-0.003 mg/mL, injection volume 5 μL. Figure S5. Representative UPLC-ESI(+)-quadrupole/orbitrap-MS chromatograms of various Neem tissue extracts (× denotes non-triterpenoids with molecular mass less than 350). Figure S6. Quantitative abundance of individual triterpenoids in different tissues of Neem. Figure S7. Multiple sequence alignment of A. indica geranyl diphosphate synthases (AiGDS). Figure S8. Multiple sequence alignment of A. indica farnesyl diphoshate synthase (AiFDS). Figure S9. Multiple sequence alignment of A. indica Squalene synthase (AiSQS); Amino acid sequence alignment of C. annuum (CaSQS, AAD20626), N. tabacum (NtSQS, AAB08578), A. indica (AiSQS, AFJ15526), L. japonicas (LjSQS, BAC56854), G. max (GmSQS, NP_001236365), P. vulgaris (PvSQS, AHA84150). The solid lines indicate four highly conserved regions 1, 2, 3 and 4 which are considered to be the catalytic sites of squalene synthases. Figure S10. Phylogenetic analysis of AiGDS, AiFDS and AiSQS. Figure S11. Purification of recombinant AiGDS, AiFDS and AiSQS. Table S1. Predicted genes for Triterpenoid back bone biosynthesis. Table S2. Present Identity Matrix of AiGDS with plant homomeric GDS and heteromeric GDS Larger subunits. Table S3. Primers and vectors used for cloning of AiGDS, AiFDS and AiSQS and RT-PCR primers of 18S rRNA, GAPDH, Neem_tran [file 12870_2015_593_MOESM1_ESM.docx]

**Additional file 1**

**Triterpenoid Profiling and Functional Characterization of the Initial Genes Involved in Isoprenoid Biosynthesis in Neem (*Azadirachta indica*)**

Avinash Pandreka,^a,b†^ Devdutta S. Dandekar,^a†^ Saikat Haldar,^a†^ Vairagkar Uttara,^a^ Shinde Vijayshree G.,^a^ Fayaj A. Mulani^a^, Thiagarayaselvam Aarthy^a^ and Hirekodathakallu V. Thulasiram^a,b,^*

^a^Chemical Biology Unit, Division of Organic Chemistry, CSIR-National Chemical Laboratory, Pune-411008, India.

^b^ CSIR-Institute of Genomics and Integrative Biology, Mall Road, New Delhi-110007, India.

^†^ Authors contributed equally.

**Methods 1: Isolation of Neem triterpenoids from seed kernel and pericarp**

The standard triterpenoids were purified and characterized as reported previously [1-4](Alam et al., 2012; Haldar et al., 2013a; Haldar et al., 2014; Haldar et al., 2013b). Extraction of dried Neem seed kernel resulted in the isolation of four major triterpenoids (Azadirachtin A, azadirachtin B, salannin, nimbin) and six minor triterpenoids (3-deacetylsalannin, 6-deacetylnimbin, nimbinene, 6-deacetylnimbinene, nimbanal, salannol acetate). Crude triterpenoid mixture was obtained by stepwise solvent partition technique. The sequential procedures of de-oiling, extraction of methanol-soluble secondary and primary metabolites followed by separation of triterpenoids by water-EtOAc partition were performed to yield the complex mixture of triterpenoids from seed kernels. Major constituents were purified by flash chromatographic technique from the crude triterpenoid mixture whereas purification of minor triterpenoids was achieved by repeated chromatographic techniques including automated MPLC, semi-preparative reverse-phase HPLC, flash chromatography and preparative TLC (Scheme 1). Crude Neem oil was also extracted and purified to isolate eight salannin and nimbin derivatives through automated MPLC technique (salannin, nimbin, 3-deacetylsalannin, 6-deacetylnimbin, nimbinene, 6-deacetylnimbinene, nimbanal, salannol acetate).

Pericarp was extracted to isolate two major triterpenoids (azadiradione and epoxyazadiradione) and three minor triterpenoids (azadirone, gedunin and nimocinol). Separation protocol was similar to the seed-kernel except slight alternation. Since the oil content in pericarp is far less than the kernel, the process of de-fatting was not essential in this case. Significant difference in polarity and less complexity of the limonoid-mixture present in the pericarp led to the excellent separation of highly pure metabolites through flash chromatographic technique. Figure 1 represents TLC of purified tritperneoids.

**Methods 2: Characterization of purified Neem triterpenoids**

Purified triterpenoids were characterized by the analyses of NMR spectrometric and HR-ESI(+)-MS data, which were in full agreement with the previously reported [1-8]. Ring-intact limonoids were characterized by the presence of C-17 furan moiety and five quaternary methyl groups at C-18, 19, 28, 29 and 30. Characterized signals in ^1^H (*δ*_H_: 7.35-7.55 for H-

Scheme 1. Solvent extraction scheme for the isolation of triterpenoids from the Neem seed kernel.

21, 7.10-7.45 for H-23 and 6.25-6.35 for H-22) and ^13^C NMR (*δ*_C_: ~142, 140, 115-125 and 110 for 23, 21, 20 and 22 respectively) spectra were utilized to identify the presence of furan

ring at C-17. Presence of five quaternary methyl groups at C-18, 19, 28, 29 and 30 were evident from ^1^H and ^13^C NMR signals within the chemical shift values ranging *δ*_H_ 0.80-1.40 and *δ*_C_ 15-25 respectively. The ubiquitous presence of an oxygenated functional group (hydroxyl or acetate in most cases) at C-7 (*δ*_H_: 4.50-5.50 and *δ*_C_: 65-75) was also observed in the ring-intact limonoid skeleton. Basic limonoids from azadirone group showed the presence of α,β-unsaturated carbonyl in the ring-A and a five membered ring-D. The presence of α,β-unsaturated carbonyl functionality was identified by the signals of two doublets (*δ*_H_: 7.10-7.20 and 5.85-5.90) in ^1^H NMR and the presence of unsaturated carbons (*δ*_C_: 155-160 and 125-126) and carbonyl (*δ*_C_: 204-205) in ^13^C NMR. The presence of six-membered lactone D-ring as observed in gedunin skeleton was confirmed by the higher chemical shift values of C-17, both in ^1^H (*δ*_H_: ~5.50) and ^13^C (*δ*_C_: ~80) NMR spectra.

C-seco limonoids of salannin and nimbin skeletons were identified by the characteristic framework of ring C/D, the presence of furan moiety at C-17 and oxygen substituted C-6 and C-7. The unsaturation (C_13-14_) in the ring C, characterized by ^13^C NMR (*δ*_C_: ~146 and 135 for C-14 and 13 respectively) spectra was a unique identity in these type of C-seco limonoid skeletons. Further, the C-D ring junction can be confirmed on the basis of signals for 15-C in ^1^H (*δ*_H_: 5.40-5.60) and ^13^C (*δ*_C_: ~87) NMR. Presence of C-7 and C-8 is indicated by the signals in ^1^H (*δ*_H_: 4.00-4.20 for H-7 and 4.00-5.20 for H-6) and ^13^C (*δ*_C_: 80-85 for C-7 and 65-75 for C-6) NMR spectra. The presence of carbonyl group at C-12 adjacent to methylene carbon (C-11) is also a common characteristic of C-seco limonoids. Nimbin type C-seco limonoid skeleton can be elucidated by the presence of α,β-unsaturated (C_2-3_) ketone functionality in ring A (*δ*_H_ of doublets: 5.85-6.05 for H-2 and 6.10-6.50 for H-3; *δ*_C_: ~201 for C-1, 125-130 for C-2, 140-150 for C-3) and a carbonyl group at C-28 (*δ*_C_ 170-205). Salannin type skeleton can be categorized by the presence of tigloyl moiety or further modified tigloyl derivative at C-1, cyclic ether ring (C-28-4-5-6) and an oxygenated substitution (acetate/hydroxyl) at C-3. In ^1^H NMR, signals for the tigloyl group appear at *δ*_H_ 6.90-7.00 (H-3*'*), 1.80-1.85 (Me-4*'*) and 1.90-1.95 (Me-5*'*). In ^13^C NMR, corresponding signals can be found in the range *δ*_C_ ~166 (C-1*'*), 128 (C-2*'*), 137-139 (C-3*'*), 10-15 (C-4*'*, 5*'*). Cyclic ether ring can be identified by the presence of C-28 through ^1^H (*δ*_H_ of doublet: 3.60-3.70) and ^13^C (*δ*_C_: ~77) NMR. Oxygen substituted C-3 appears at *δ*_H_ 3.75-5.00 and *δ*_C_ ~70. Pentanortriterpenoids of nimbinene skeleton can be characterized by the removal of Me-28 and the repositioning of unsaturation (C_3-4_) (*δ*_H_ 5.40-5.50 for H-3; *δ*_C_ ~120 for C-3 and 135-140 for C-4) in ring A.

Azadirachtin skeleton is characterized by the typical construction of rearranged C/D and furan ring. The unsaturation at C_22-23_ is identified by the signals at *δ*_H_ ~6.45 (d, H-23) and 5.03 (d, H-22) in ^1^H NMR. ^13^C NMR chemical shift values (*δ*_C_ ~147 for C-23 and 107 for C-22) also support the presence of C_22-23_ double bond. The existence of characteristic ring junction at C-20-21 can be identified by the signal at *δ*_H_ ~5.65 (H-21) and *δ*_C_ ~108 (C-21), 83 (C-20). The presence of cyclic five-membered ether ring (C-11-9-10-19) can be identified by the higher chemical shift values for C-11 (*δ*_C_ 75-105) and C-19 (*δ*_H_ 4.00-4.20; *δ*_C_ ~70). Ring A and B contain various characteristic functional groups (similar to salannin skeleton) such as tigloyl, esters and hydroxyls with variability in their positions creating a large number of azadirachtin derivatives. The presence of cyclic ether joining the rings A/B (as in salannin type limonoid) and 7-hydroxyl (as in azadirone type limonoid) is a common structural feature of azadirachtin skeleton.

***
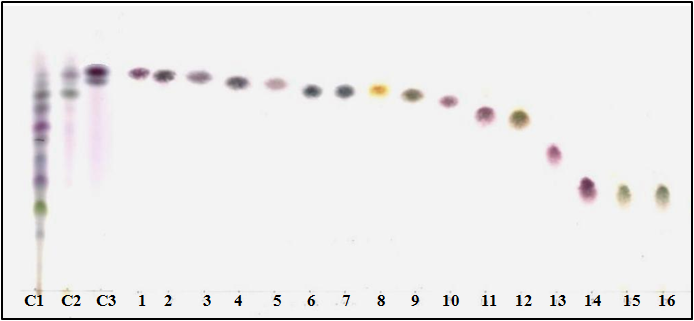
***

Figure S1. TLC profile of crude extracts and purified triterpenoids (developed in 70% ethyl acetate in *n*-hexane for twice).

C1- Neem seed kernel extract

C2- Neem fruit extract

C3- Neem flower extract

1- Azadirone

2- Nimbinene

3- Epoxyazadiradione

4- Nimocinol

5- Gedunin

6- Nimbanal

7- 6-Deacetylnimbinene

8- 17β-Hydroxyazadiradione

9- Azadiradione

10- Nimbin

11- 6-Deacetylnimbin

12- Salannol acetate

13- Salannin

14- 3-Deacetylsalannin

15- Azadirachtin B

16- Azadirachtin A

***References***

1. Alam, A., Haldar, S., Thulasiram, H.V., Kumar, R., Goyal, M., Iqbal, M.S., Pal, C., Dey, S., Bindu, S., Sarkar, S., et al. Novel anti-inflammatory activity of epoxyazadiradione against macrophage migration inhibitory factor: Inhibition of tautomerase and proinflammatory activities of macrophage migration inhibitory factor. *J Biol Chem*. 2012;287:24844-61.
2. Haldar, S., Kolet, S.P., and Thulasiram, H.V. Biocatalysis: fungi mediated novel and selective 12β- or 17β-hydroxylation on the basic limonoid skeleton. *Green Chem*. 2013;15:1311-7.
3. Haldar, S., Mulani, F.A., Aarthy, T., Dandekar, D.S., and Thulasiram, H.V. Expedient preparative isolation and tandem mass spectrometric characterization of C-seco triterpenoids from Neem oil. *J Chromatogr A*. 2014;1366:1-14.
4. Haldar, S., Phapale, P.B., Kolet, S.P., and Thulasiram, H.V. Expedient preparative isolation, quantification and characterization of limonoids from Neem fruits. *Anal Methods*. 2013;5**:**5386-91.
5. Rojatkar, S.R., Bhat, V.S., Kulkarni, M.M., Joshi, V.S., and Nagasampagi, B.A. Tetranortriterpenoids from *Azadirachta indica*. *Phytochemistry*. 1989;28:203-5.
6. Johnson, S., and Morgan, E.D. Comparison of chromatographic systems for triterpenoids from Neem (*Azadirachta indica*) seeds. *J Chromatogr A*. 1997;761: 53-63.
7. Suresh, G., Narasimhan, N.S., and Palani, N. Structure of nimonol from fresh whole green leaves of *Azadirachta indica*. *Phytochemistry*. 1997;45: 807-10.
8. Kraus, W., and Cramer, R. Pentanortriterpenoide aus *Azadirachta indica* A. Juss (Meliaceae). *Chem Ber*. 1981;114:2375-81.


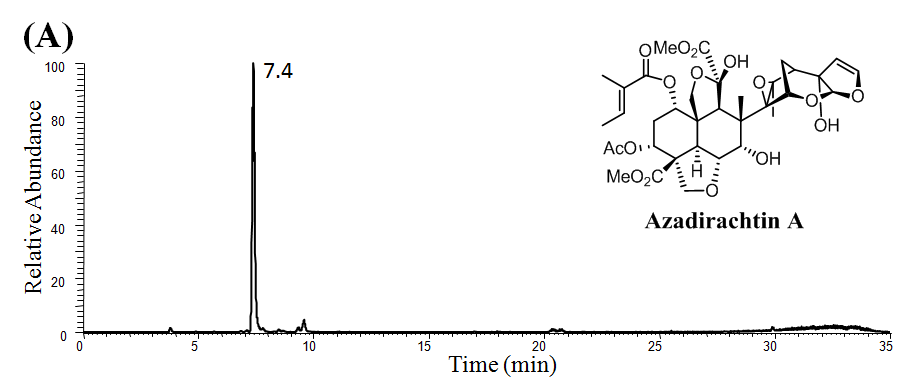

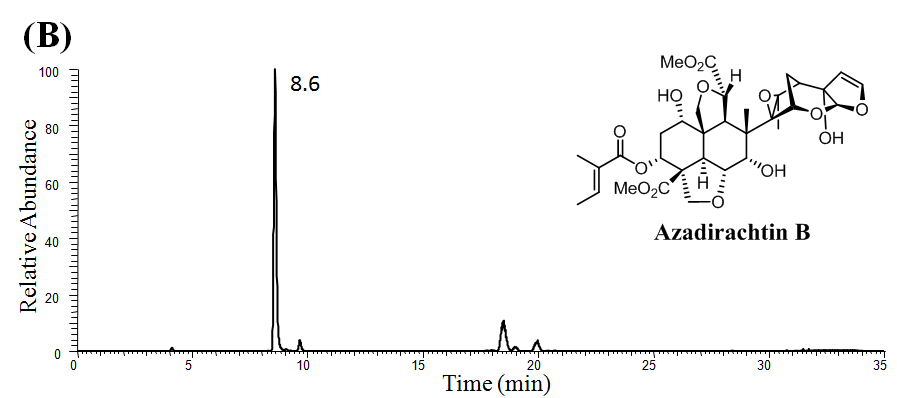


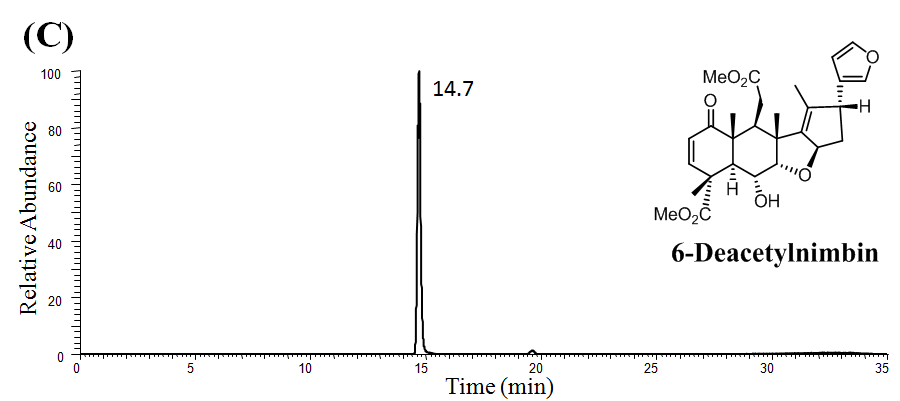

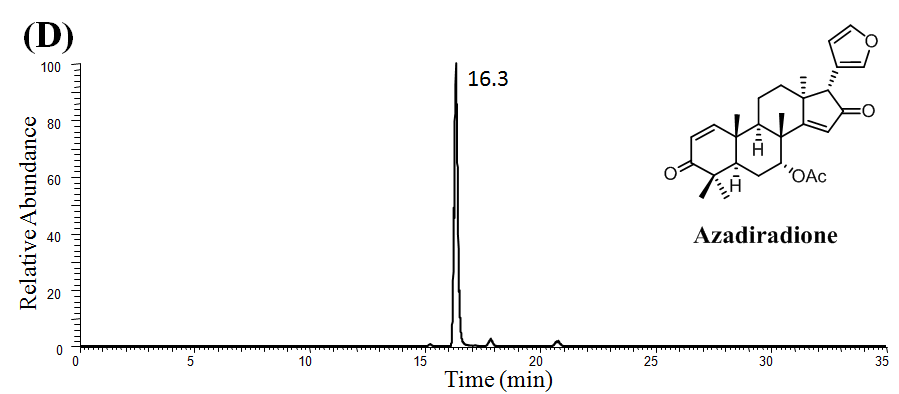


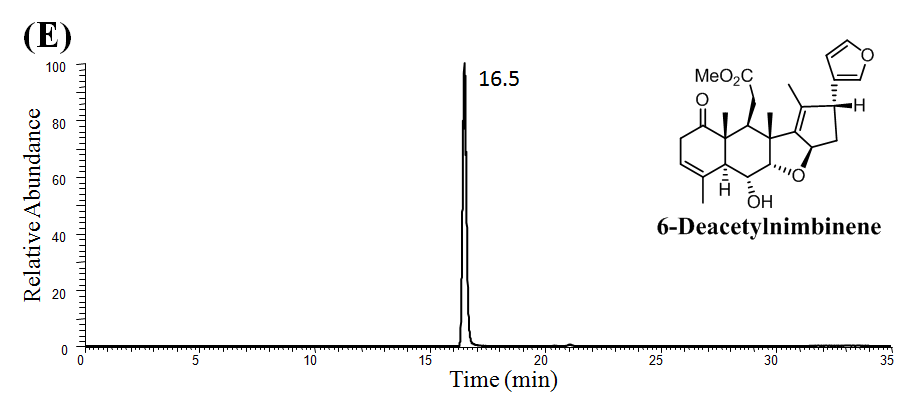

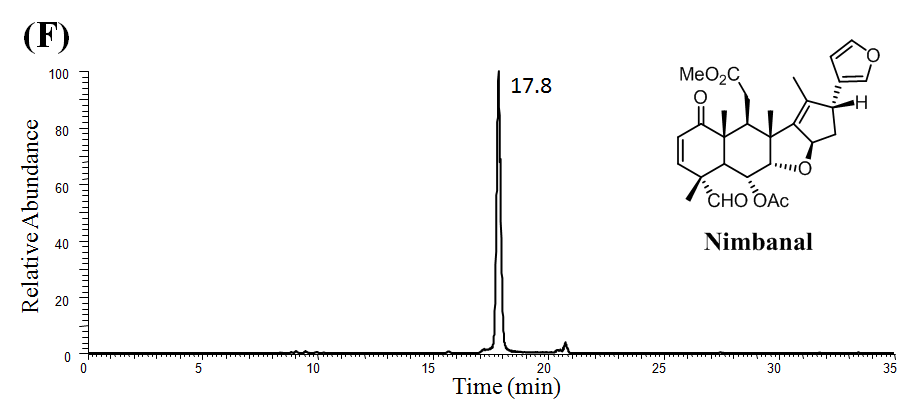


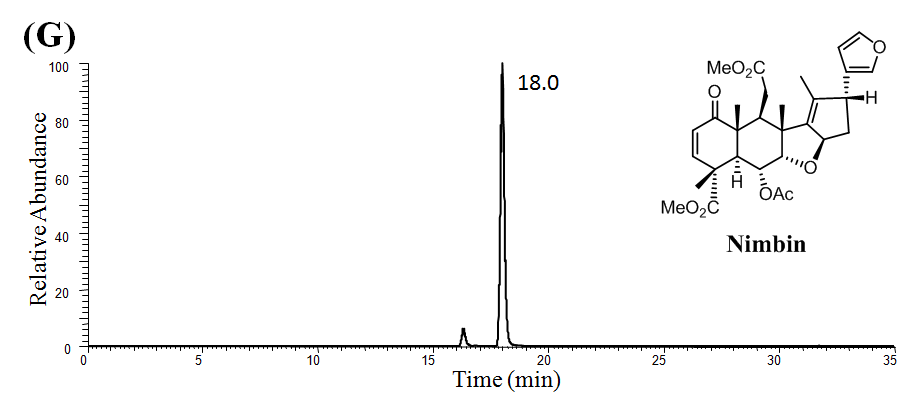

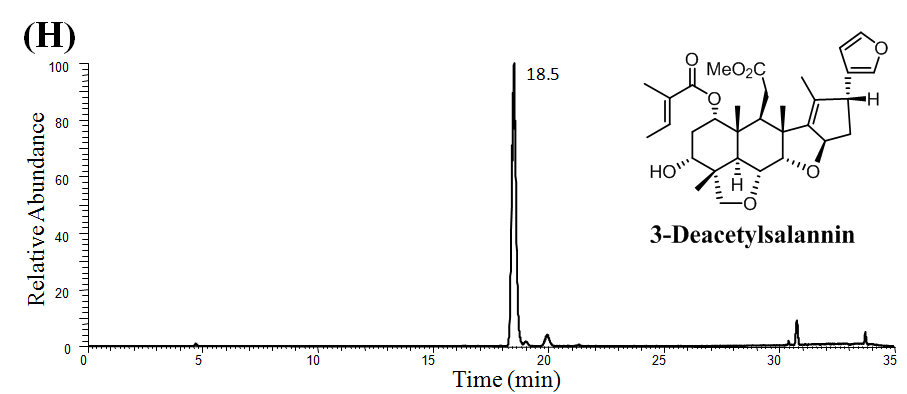


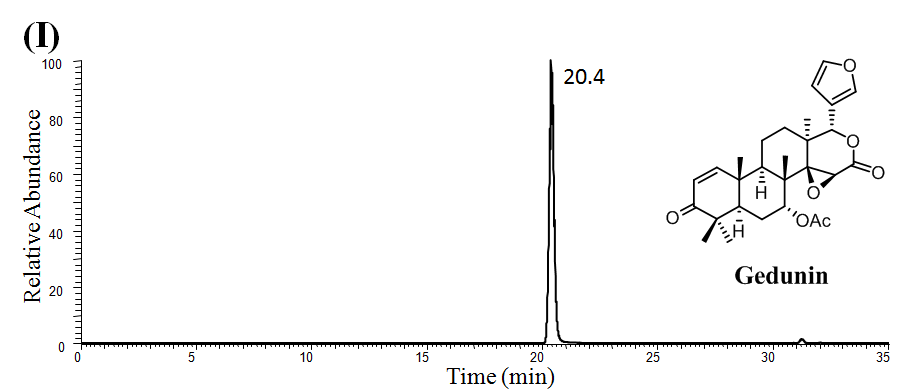

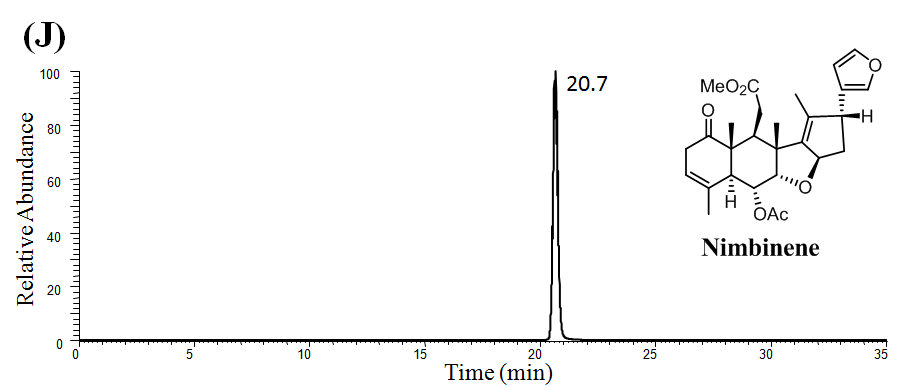


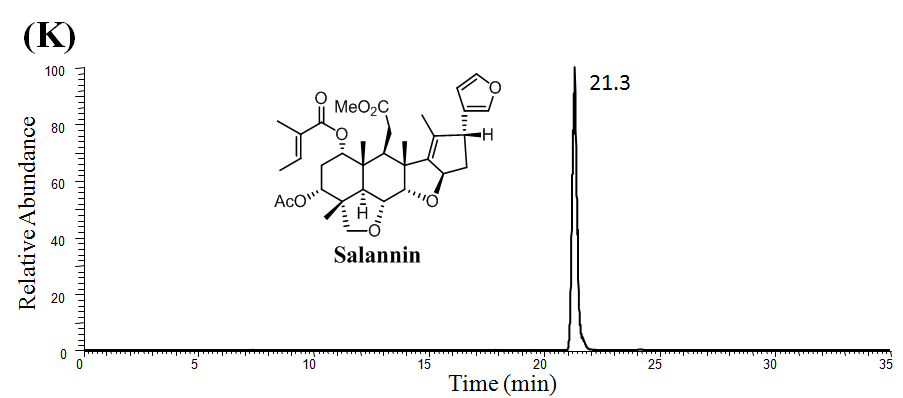

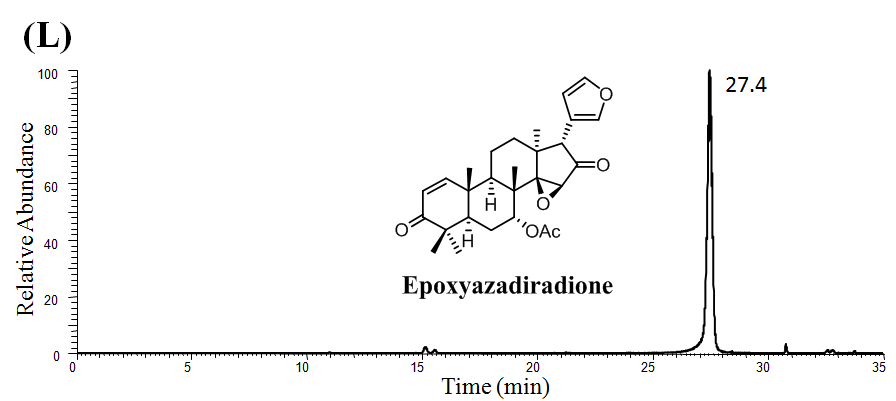


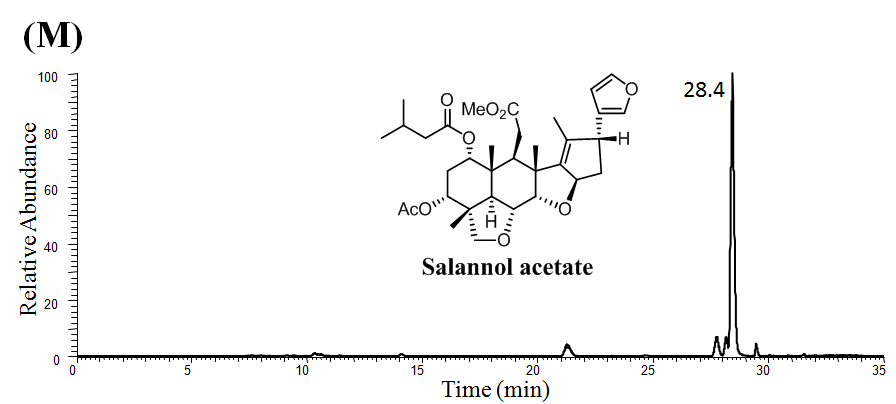

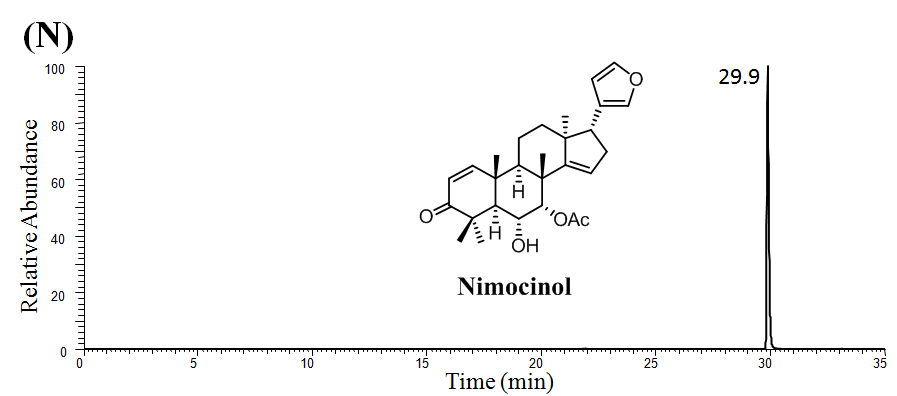


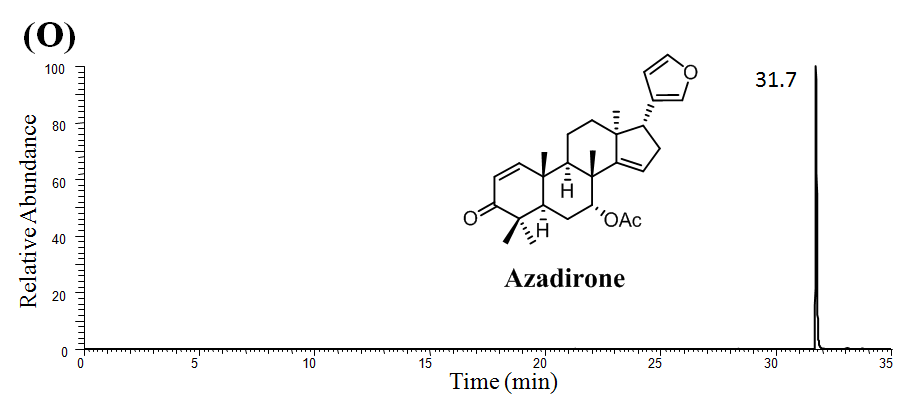


Figure S2. UPLC-ESI(+)-quadrupole/orbitrap-MS extracted ion chromatograms of the fifteen pure triterpenoids from Neem; (A) Azadirachtin A, (B) Azadirachtin B, (C) 6-Deacetylnimbin, (D) Azadiradione, (E) 6-Deacetylnimbinene, (F) Nimbanal, (G) Nimbin, (H) 3-Deacetylsalannin, (I) Gedunin, (J) Nimbinene, (K) Salannin, (L) Epoxyazadiradione, (M) Salannol acetate, (N) Nimocinol, (O) Azadirone. Chromatograms have been arranged in the order of increasing retention time.


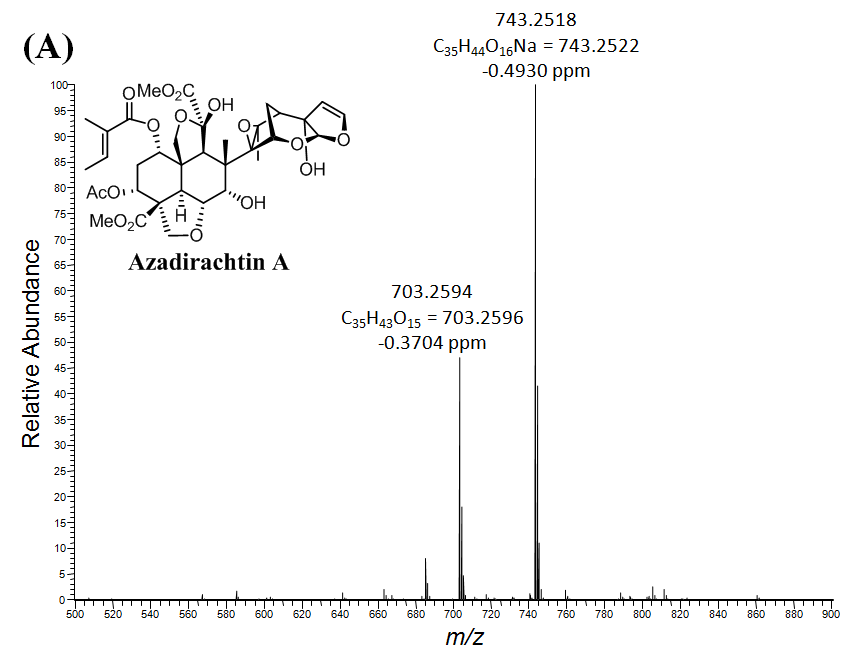

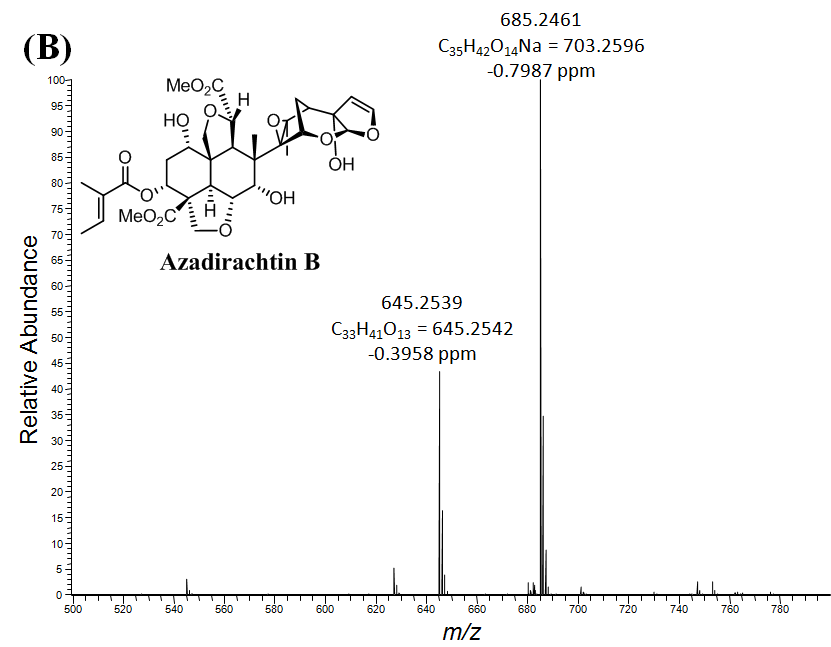


**
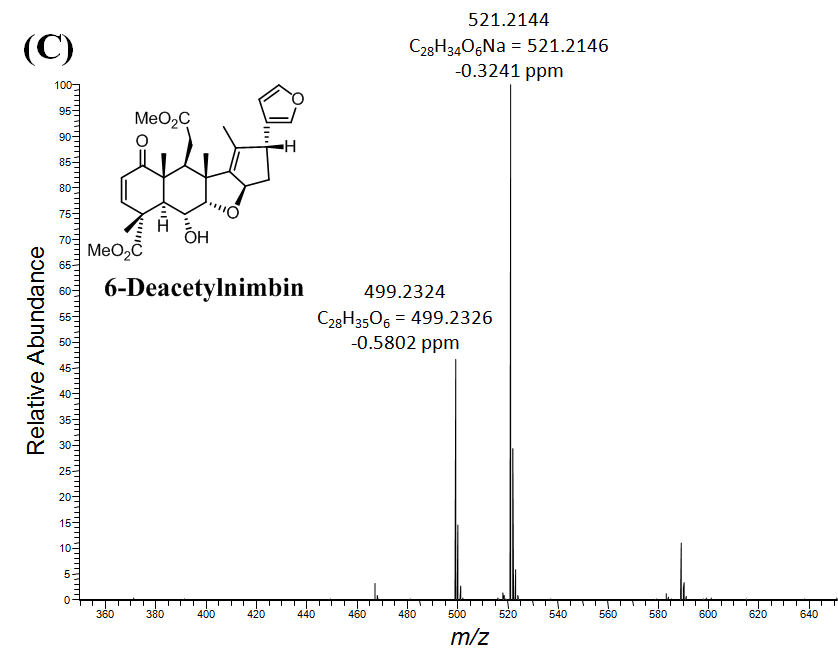

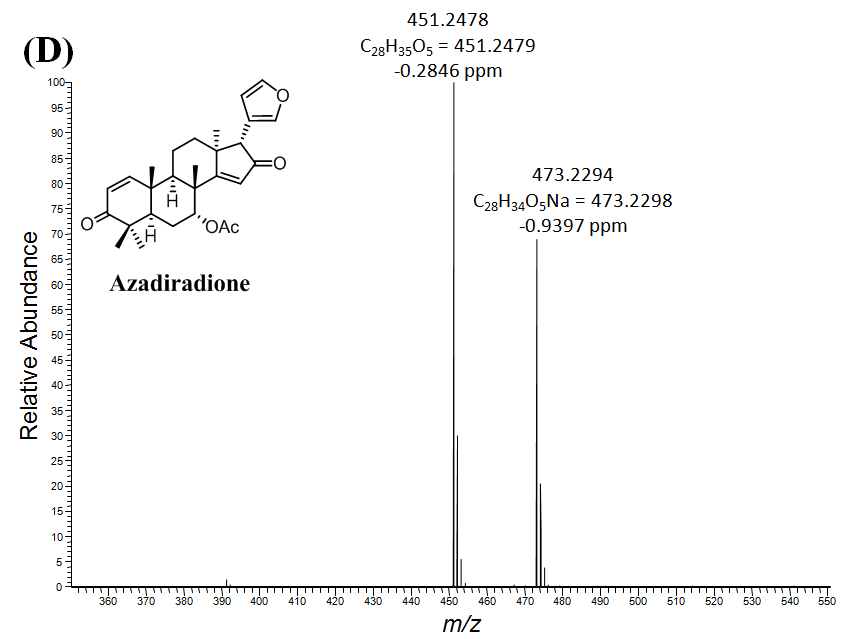
**

**
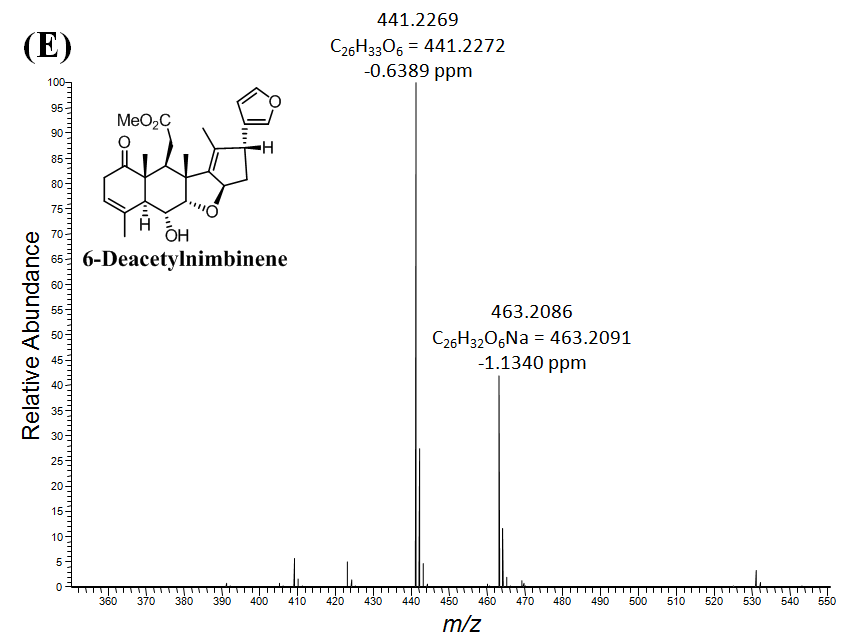

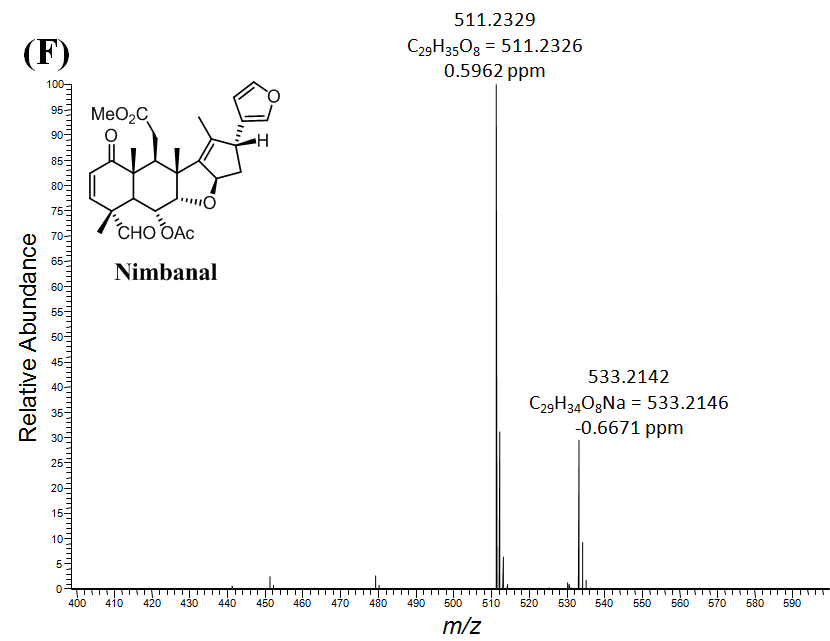
**

**
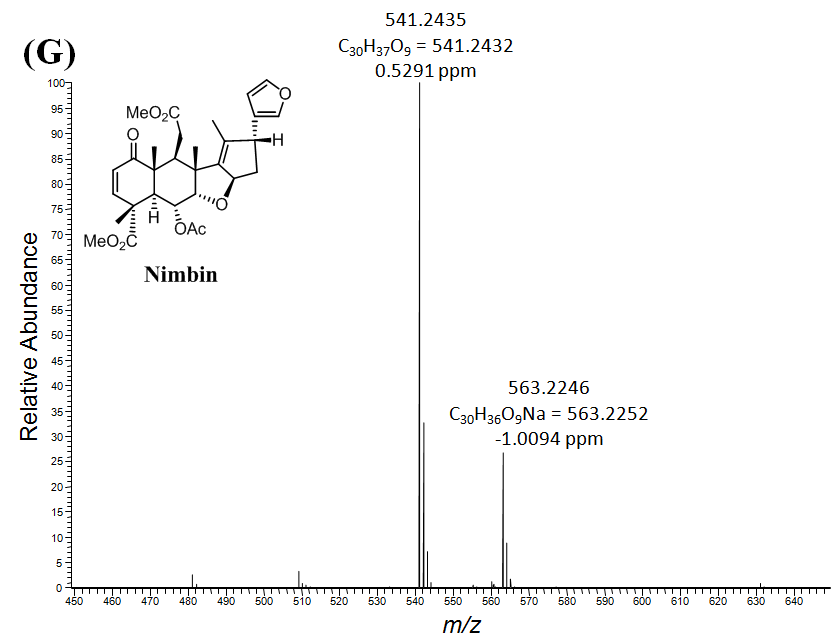

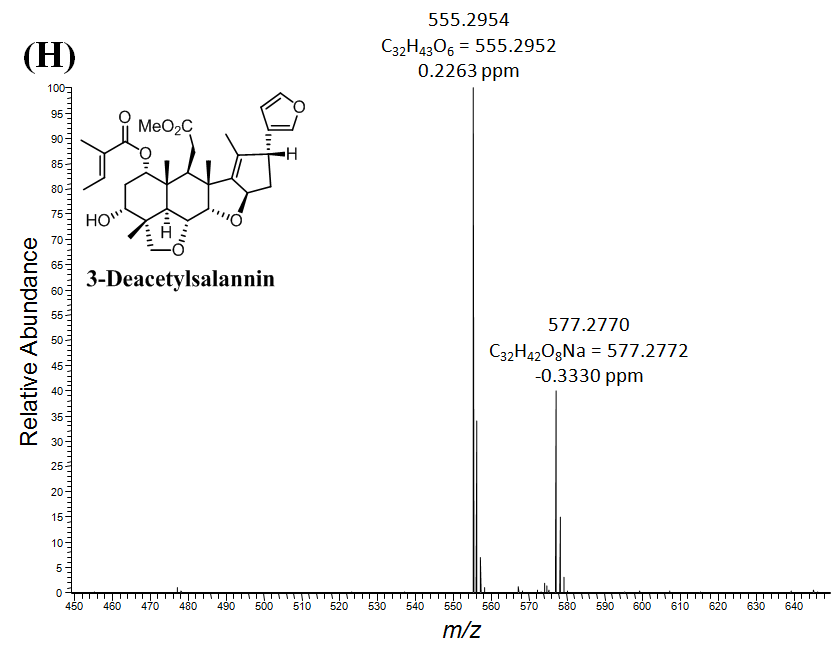
**

**
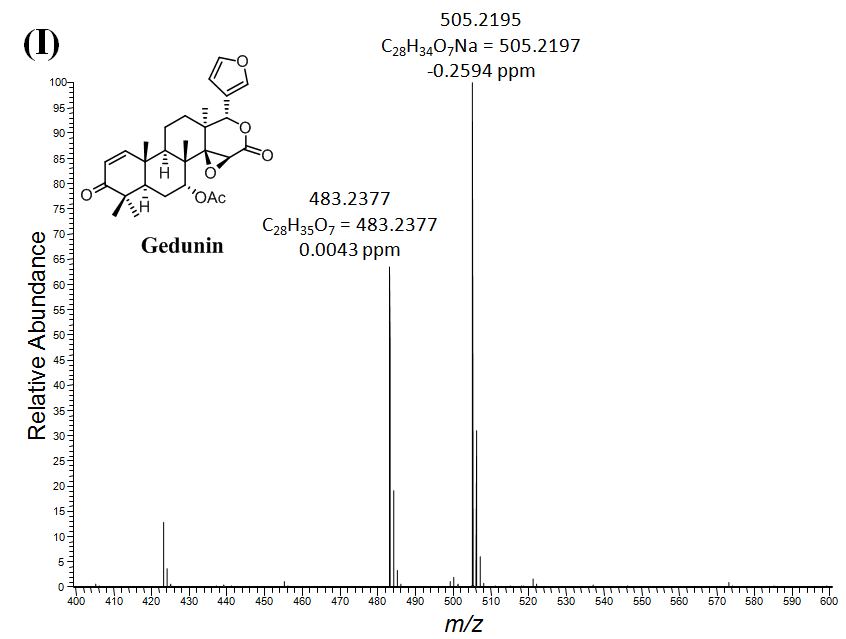

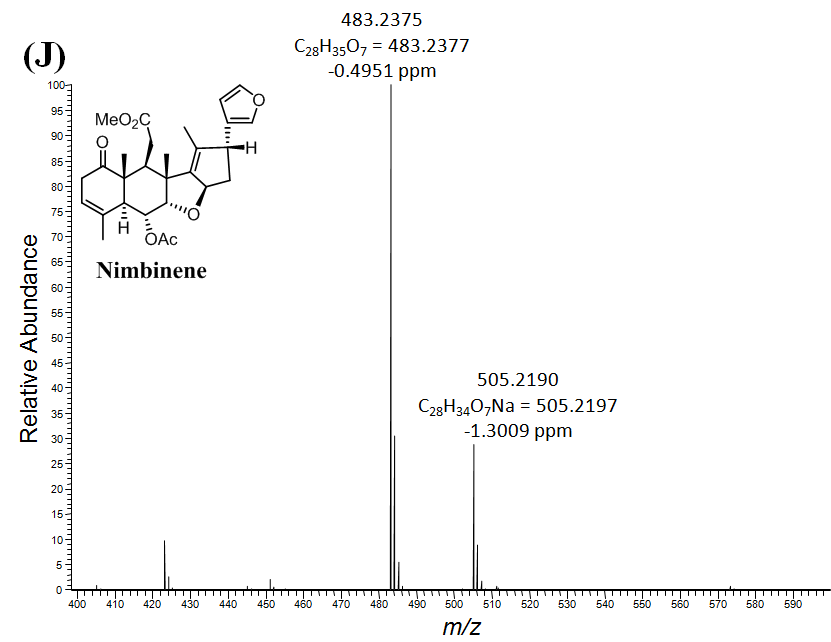
**

**
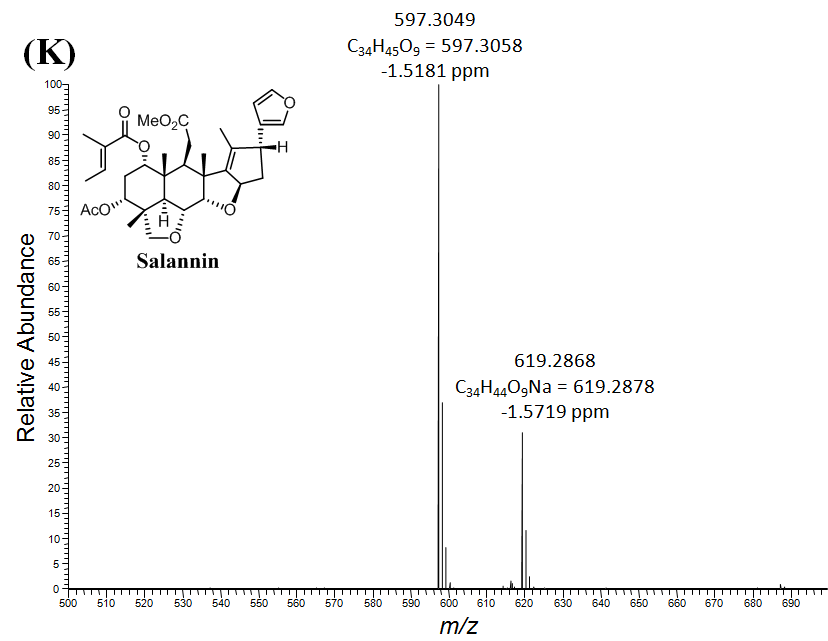

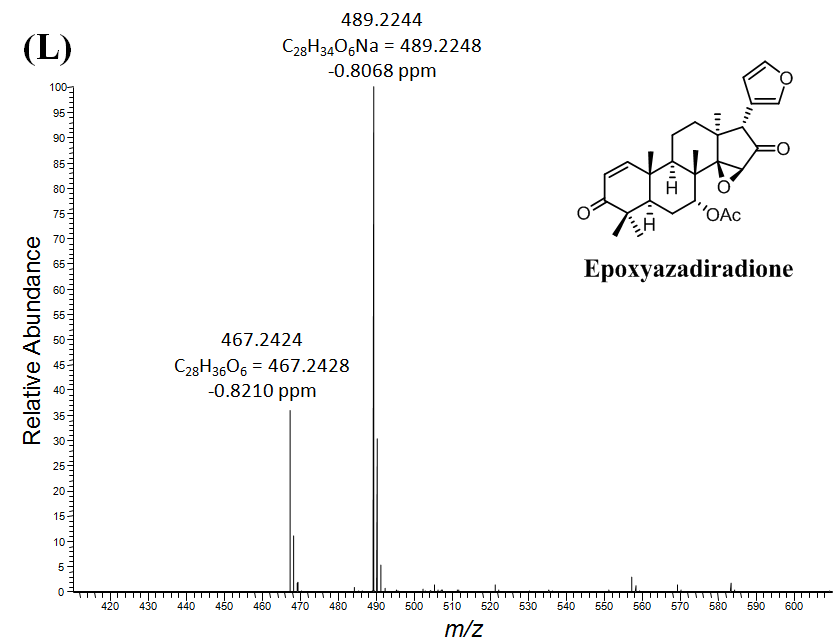
**

**
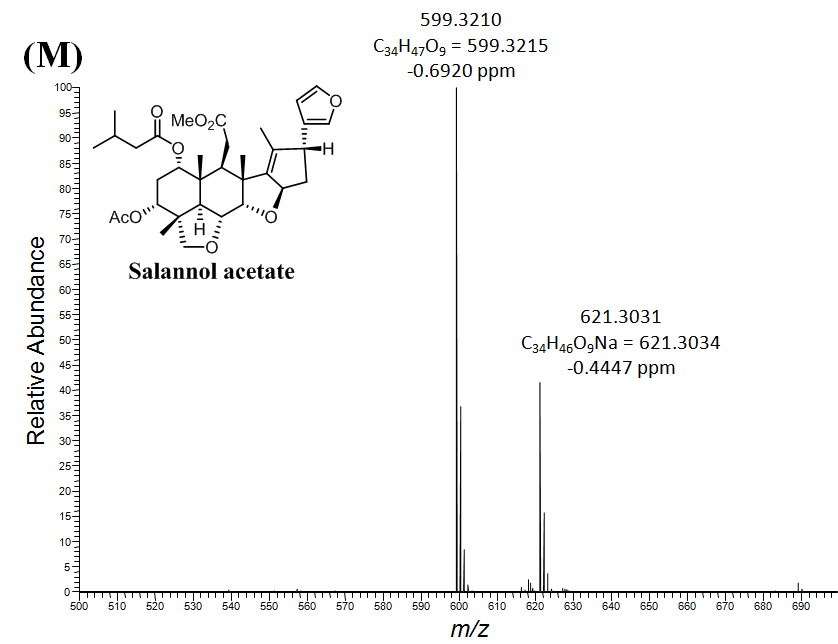

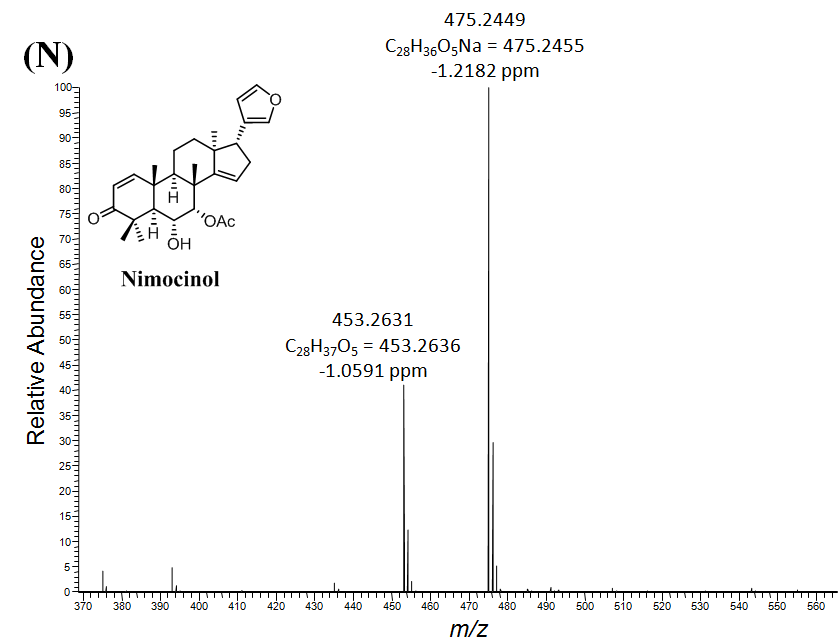
**

**
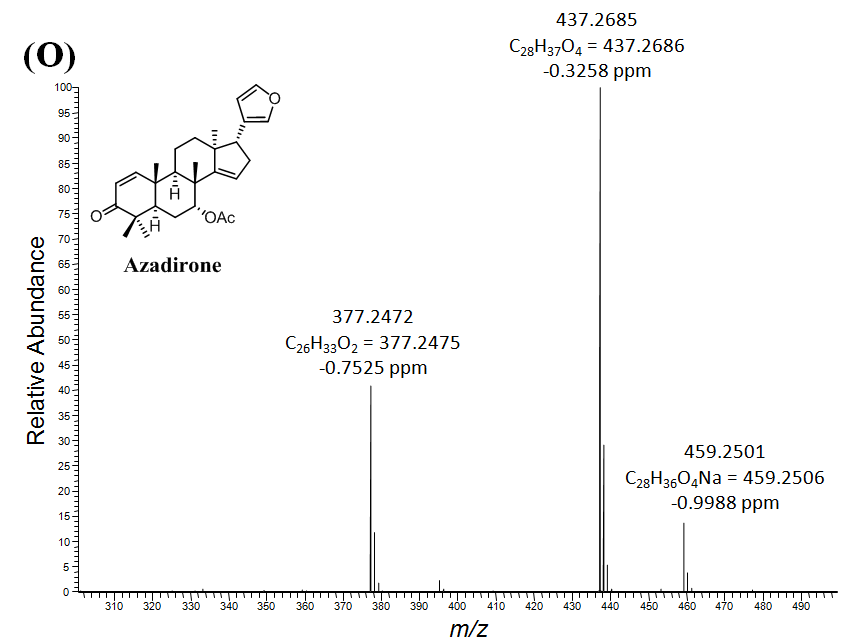
**

Figure S3. ESI(+)-quadrupole/orbitrap-MS spectra of the fifteen pure triterpenoids from Neem; (A) Azadirachtin A, (B) Azadirachtin B, (C) 6-Deacetylnimbin, (D) Azadiradione, (E) 6-Deacetylnimbinene, (F) Nimbanal, (G) Nimbin, (H) 3-Deacetylsalannin, (I) Gedunin, (J) Nimbinene, (K) Salannin, (L) Epoxyazadiradione, (M) Salannol acetate, (N) Nimocinol, (O) Azadirone.

|  |  |  |
| --- | --- | --- |
|  |  |  |
|  |  |  |
|  |  |  |
|  |  |  |

Figure S4. Standard graphs for the purified triterpenoids prepared in UPLC-ESI(+)-quadrupole/orbitrap-MS; concentration range 0.040-0.003 mg/mL, injection volume 5 μL.

**
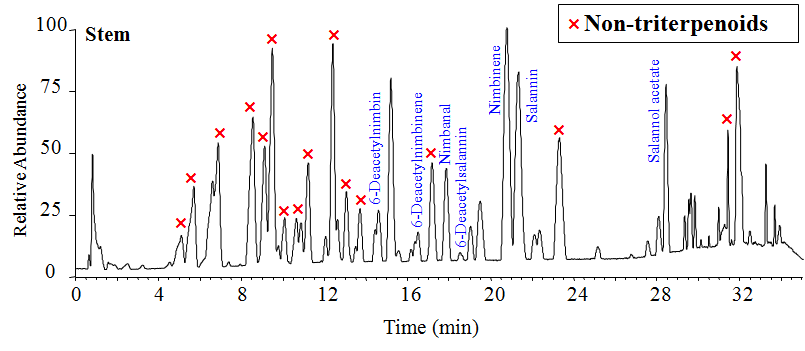

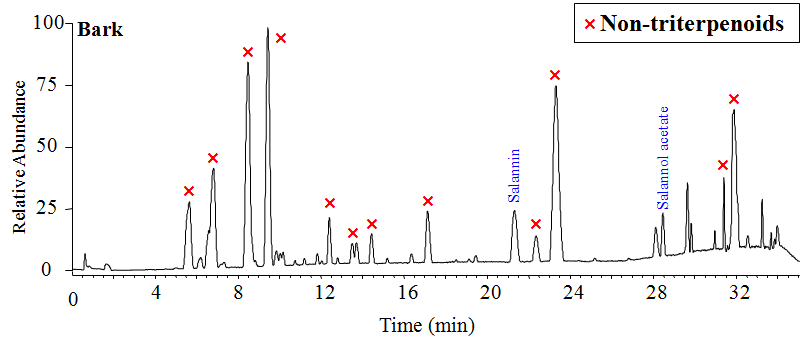

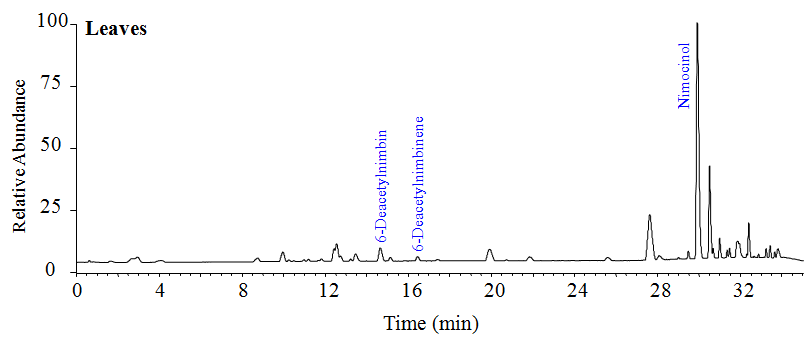

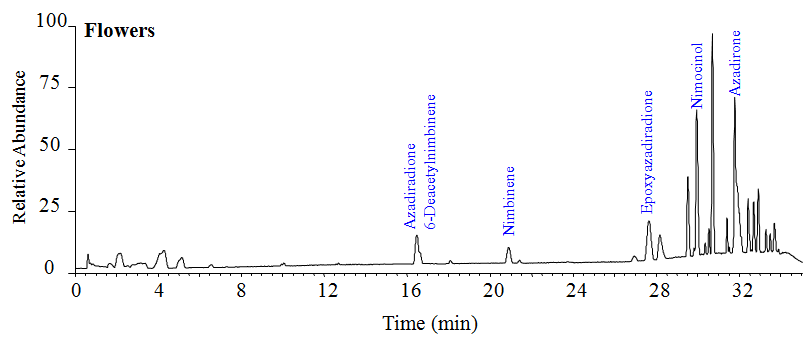
**

**
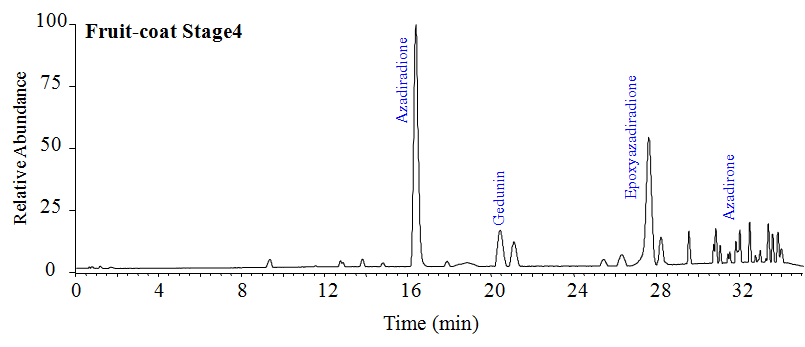

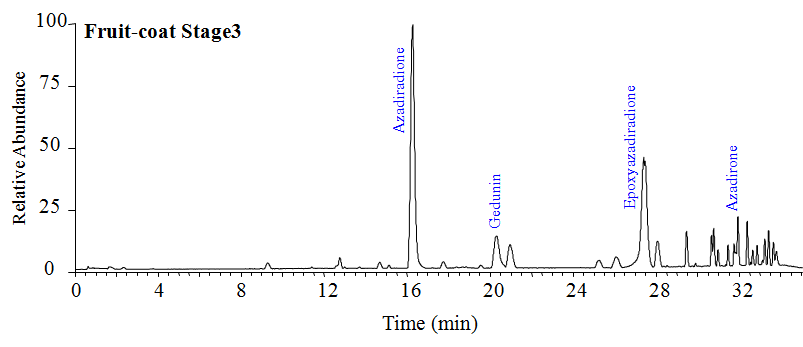

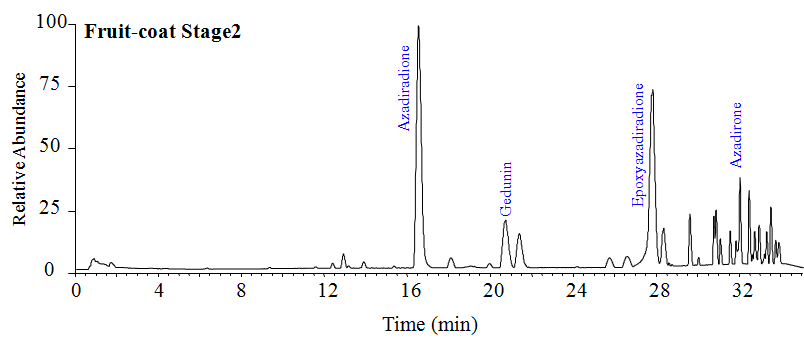

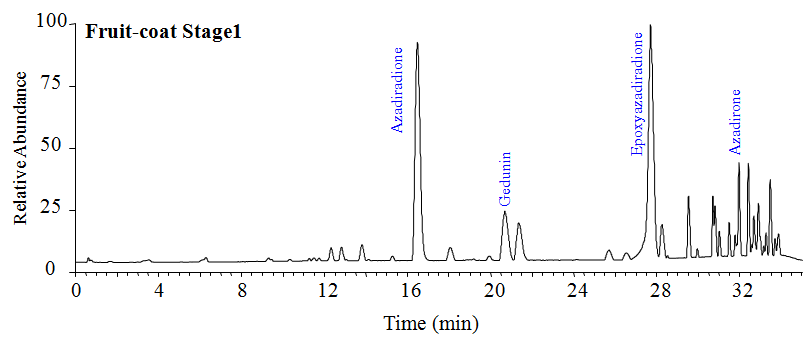
**

**
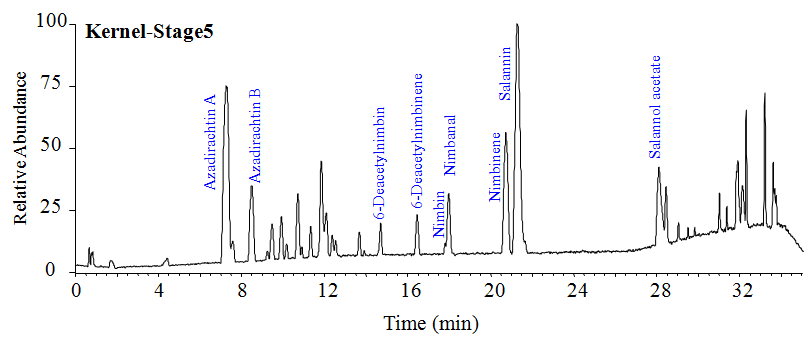

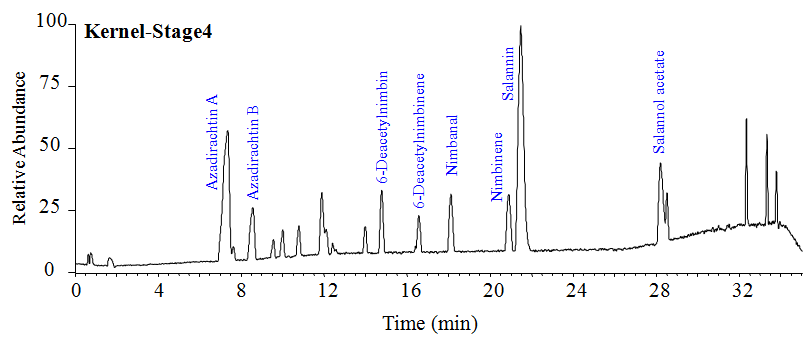

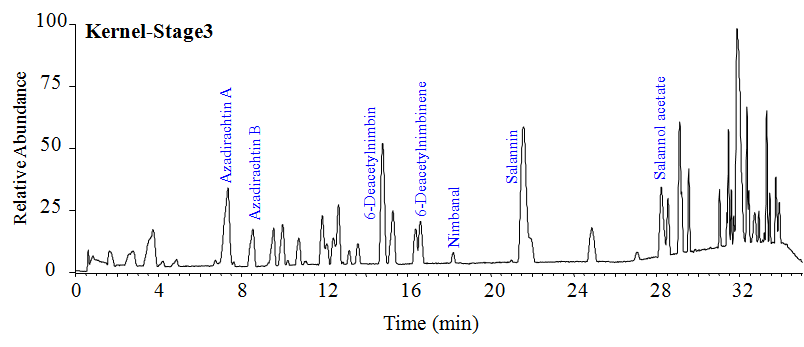

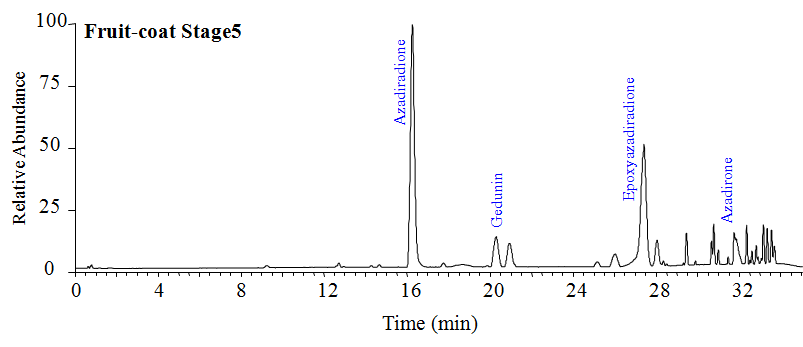
**

Figure S5. Representative UPLC-ESI(+)-quadrupole/orbitrap-MS chromatograms of various Neem tissue extracts (× *denotes non-triterpenoids with molecular mass less than 350*).

| 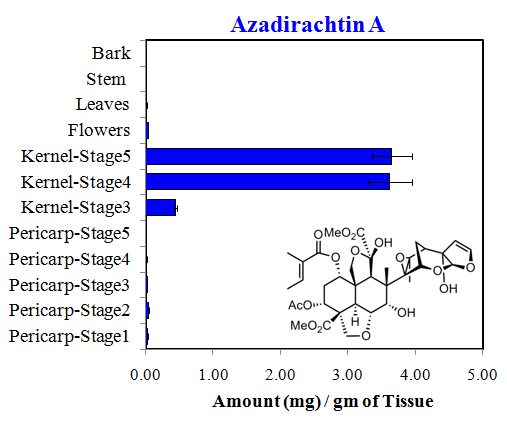 | 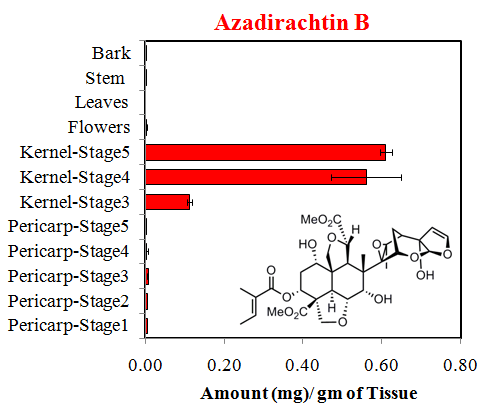 | 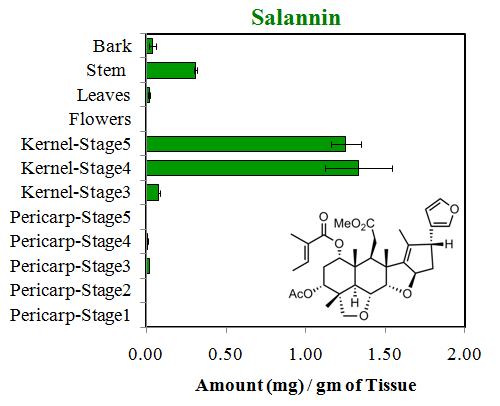 |
| --- | --- | --- |
| 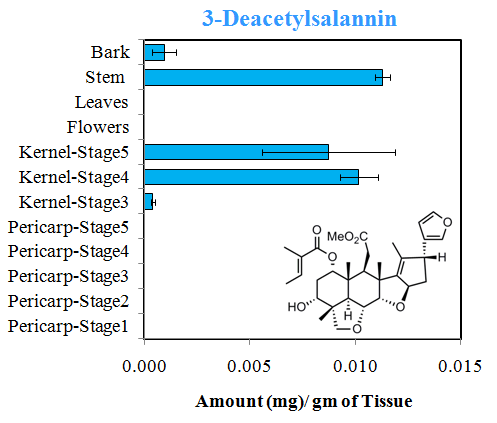 | 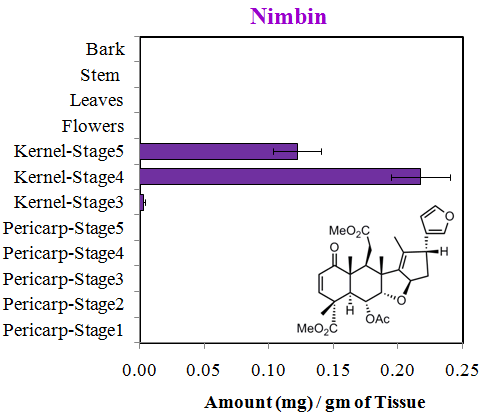 | 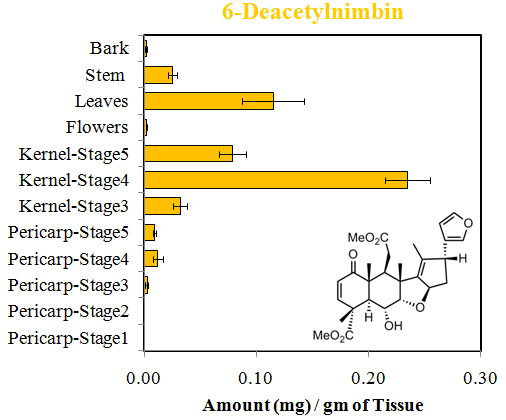 |
| 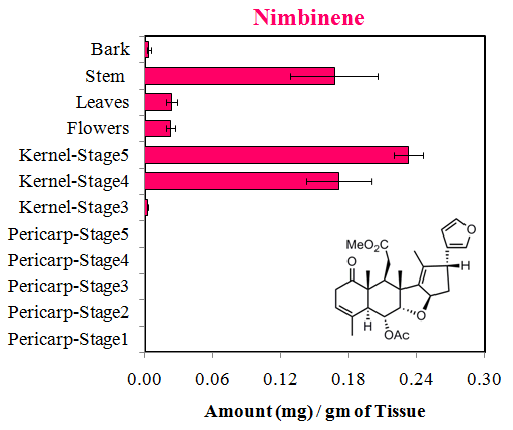 | 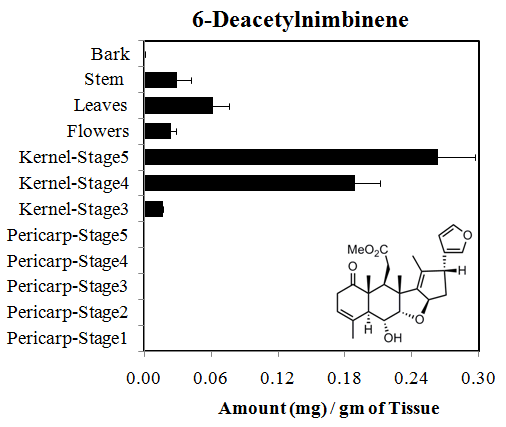 | 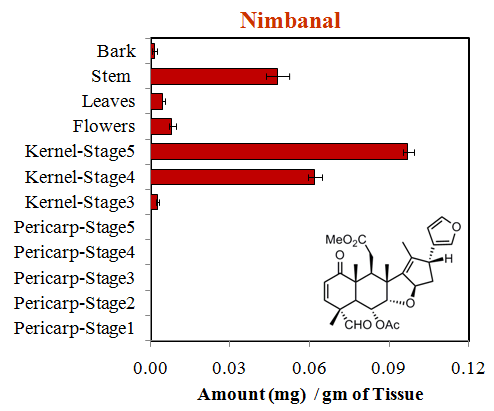 |
| 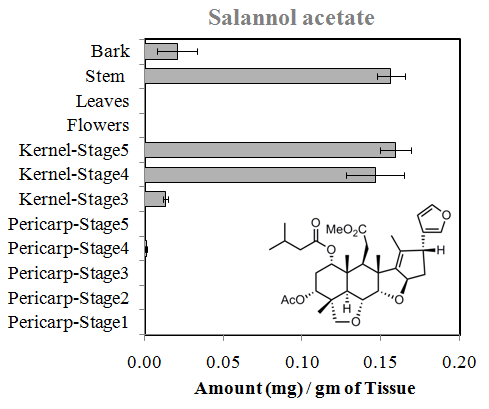 | 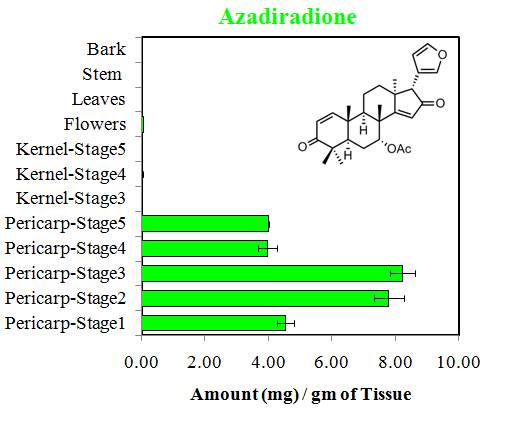 | 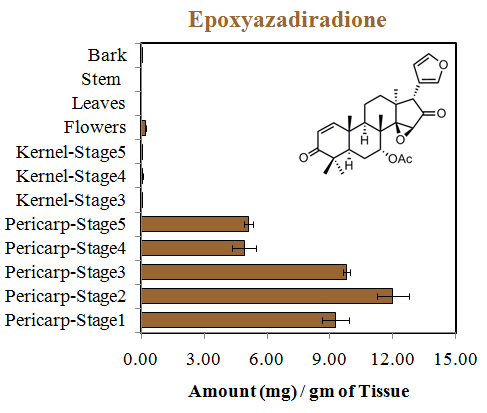 |
| 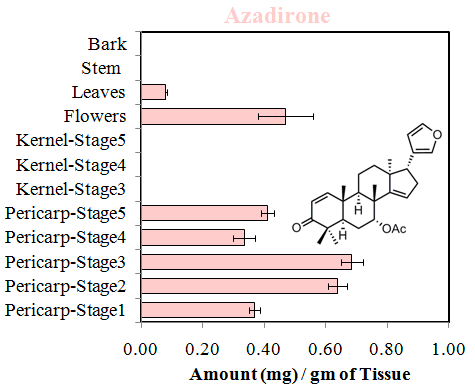 | 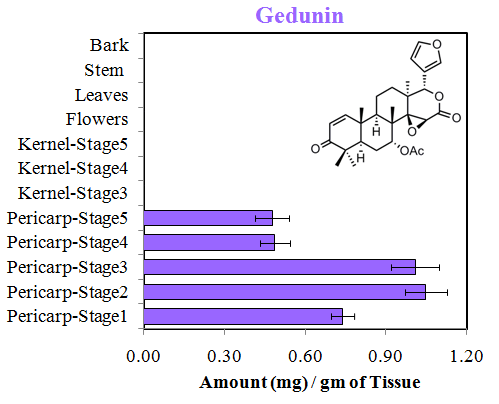 | 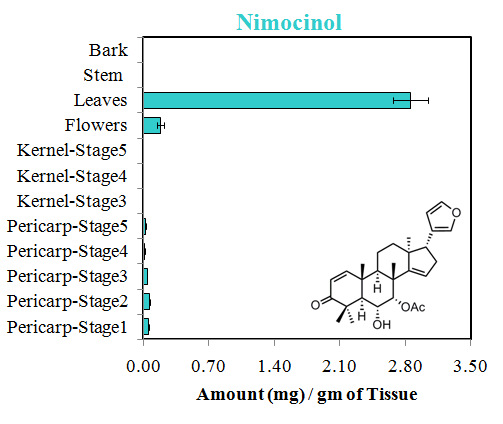 |

Figure S6. Quantitative abundance of individual triterpenoids in different tissues of Neem.


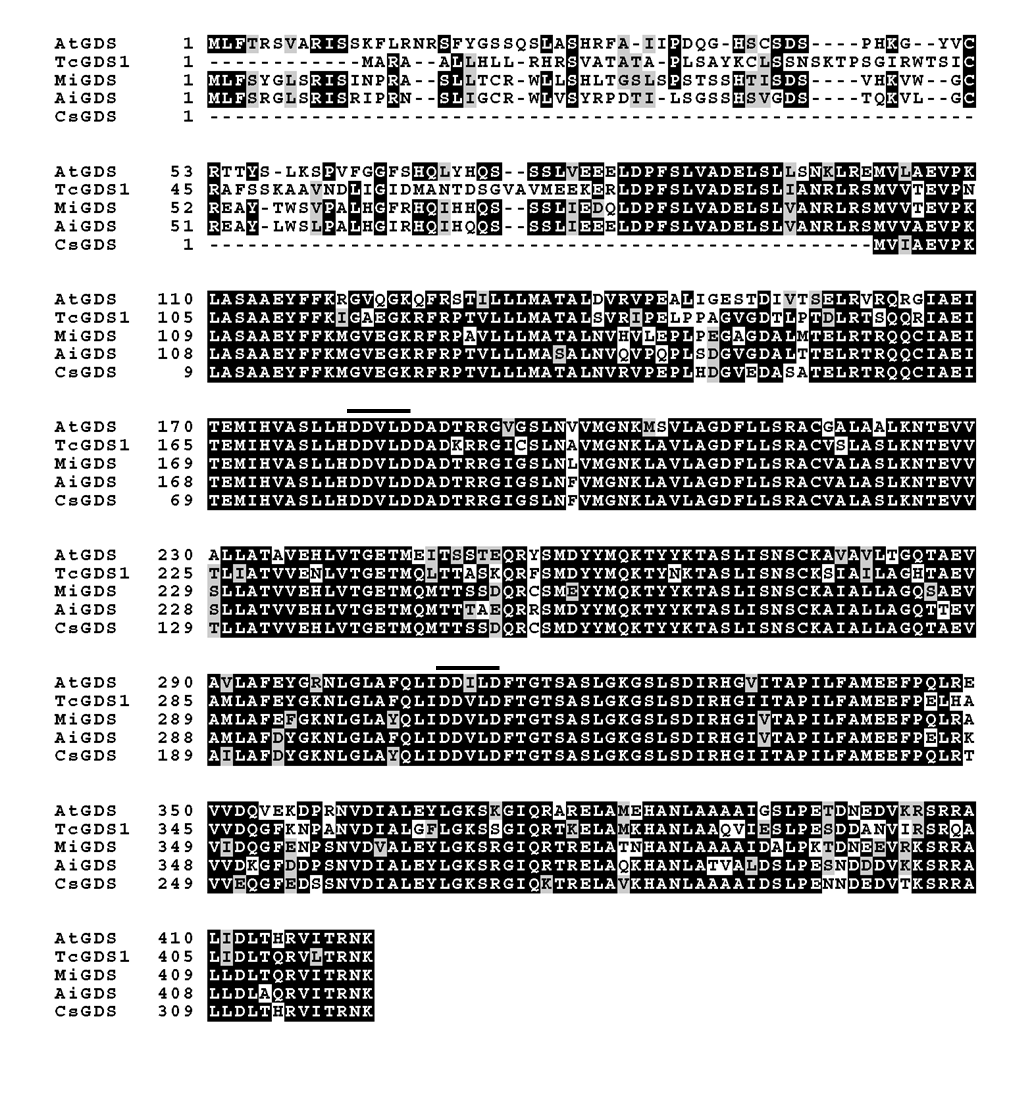


Figure S7. Multiple sequence alignment of *A. indica* geranyl diphosphate synthases (AiGDS); Amino acid sequence alignment of *T. cacao* (TcGDS_1, XP_007016031), *A. thaliana* (AtGDS, CAC16849), *M. indica* (MiGDS), *A. indica* (AiGDS) and *C. sinensisi* (CsGDS, CAC16851). The highly conserved Asp-rich motifs of prenyltransferases are indicated by solid line.


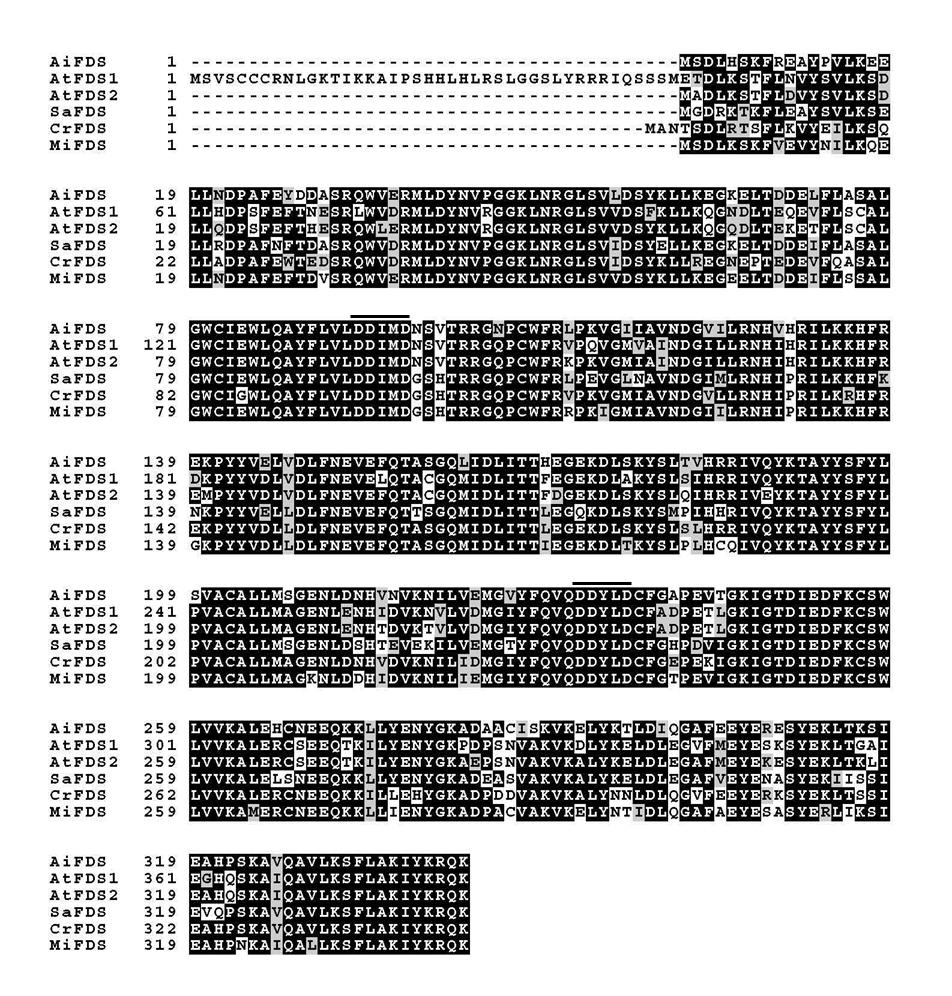


Figure S8. Multiple sequence alignment of *A. indica* farnesyl diphoshate synthase (AiFDS); Amino acid sequence alignment of *A. Thaliana* (AtFDS1, NP_199588), *A. Thaliana* (AtFDS2, AAB07248), *S. album* (SaFDS, AEY80378), *C. roseus* (CrFDS, ADO95193), *A. indica* (AiFDS, KM108316) and *M. indica* (AFJ52720). The highly conserved Asp-rich motifs of prenyltransferases are indicated by solid line.


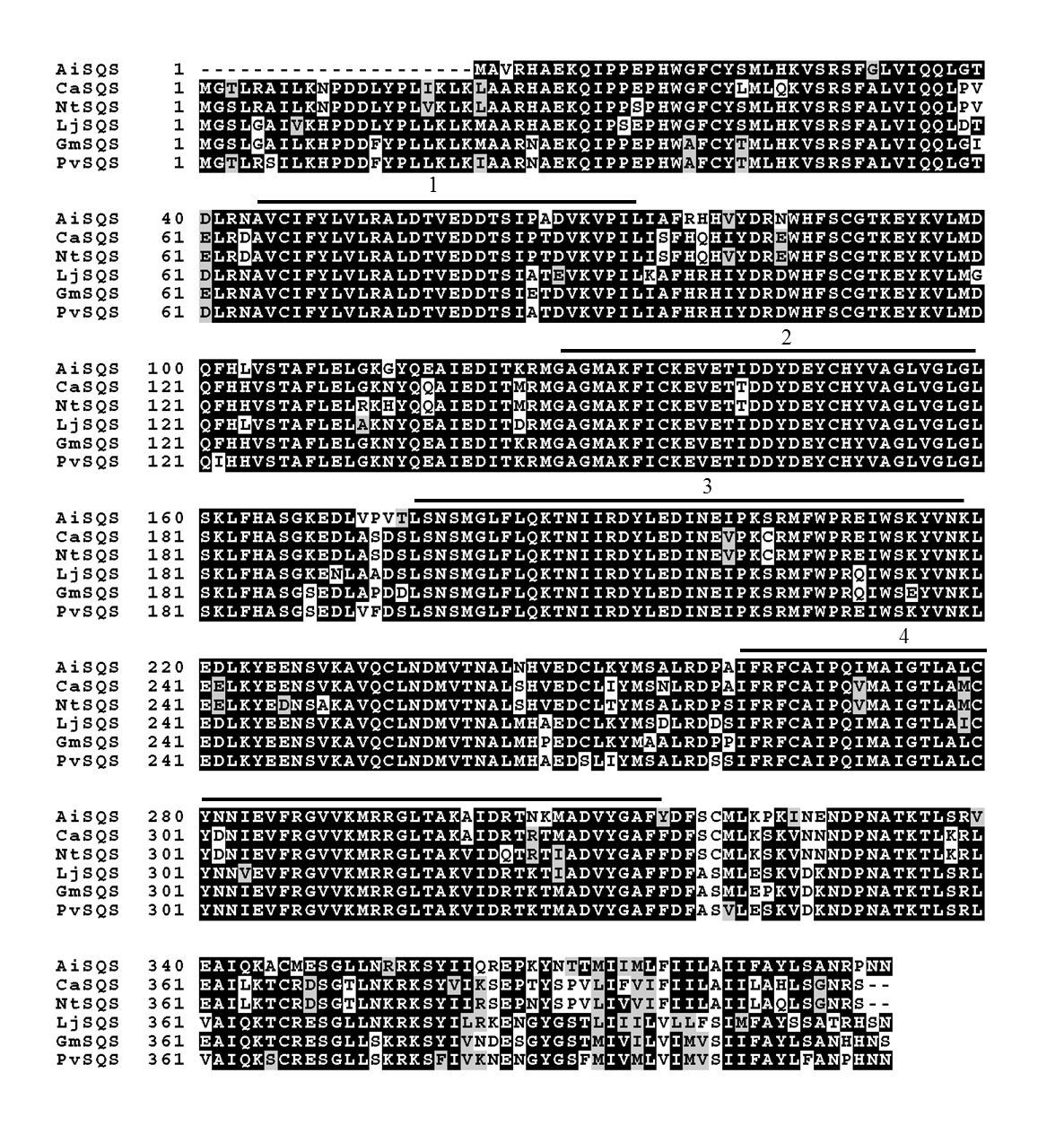


Figure S9. Multiple sequence alignment of *A. indica* Squalene synthase (AiSQS); Amino acid sequence alignment of *C. annuum* (CaSQS, AAD20626), *N. tabacum* (NtSQS, AAB08578), *A. indica* (AiSQS, AFJ15526), *L. japonicas* (LjSQS, BAC56854), *G. max* (GmSQS, NP_001236365), *P. vulgaris* (PvSQS, AHA84150). The solid lines indicate four highly conserved regions 1, 2, 3 and 4 which are considered to be the catalytic sites of squalene synthases.


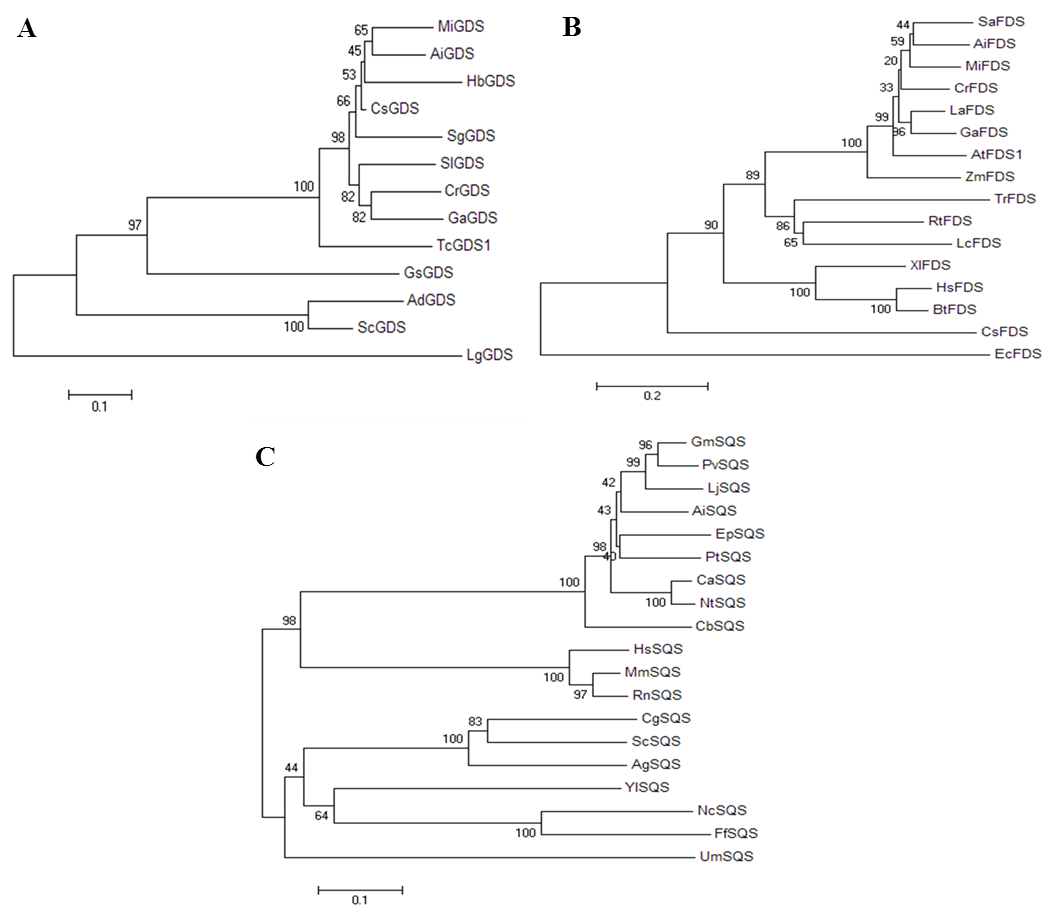


Figure S10. Phylogenetic analysis of AiGDS, AiFDS and AiSQS; (A) Phylogenetic tree of the deduced amino acid sequences of AiGDS with GDS from different organisms. (B) Phylogenetic tree of the deduced amino acid sequences of AiFDS with FDS from different organisms. (C) Phylogenetic tree of the deduced amino acid sequences of AiSQS with SQS from different organisms.


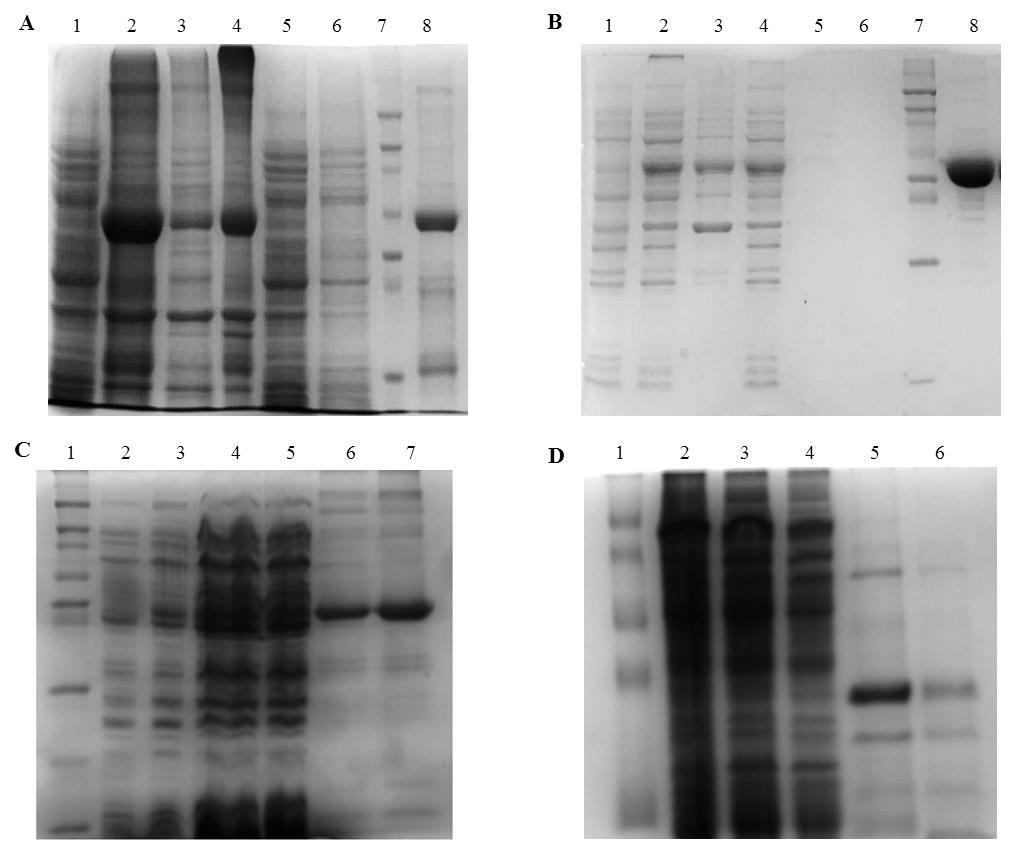


Figure S11. Purification of recombinant AiGDS, AiFDS and AiSQS; (A) SDS gel picture showing 66 kDa purified AiGDS which was expressed in Lemo 21(DE3) cells. (Lane 1) Supernatant, (Lane 2) Pellet, (Lane 3) Supernatant of pH adjustment, (Lane 4) Pellet of pH adjustment, (Lane 5) Unbound fraction, (Lane 6) Wash fraction, (Lane 7) Novex® Sharp Pre-stained Protein Standard, (Lane 8) 66 kDa purified AiGDS. (B) SDS gel picture showing 59.5 kDa purified AiFDS which was expressed in BL21 (DE3) cells. (Lane 1) Supernatant, (Lane 2) Pellet, (Lane 3) Unbound fraction, (Lane 4) Wash fraction 1, (Lane 5) Wash fraction 2, (Lane 6) Wash fraction 3, (Lane 7) Novex® Sharp Pre-stained Protein Standard, (Lane 8) 59.5 kDa purified AiFDS. (C) SDS gel picture showing 44 kDa purified full length AiSQS which was expressed BL21 Star (DE3) cells. (Lane 1) Novex® Sharp Pre-stained Protein Standard, (Lane 2) Supernatant, (Lane 3) Pellet, (Lane 4) Unbound fraction, (Lane 5) Wash fraction, (Lane 6 and 7) 44 kDa purified AiSQS. (D) SDS gel picture showing 35 kDa purified truncated AiSQS which was expressed BL21 (DE3) cells. (Lane 1) Bangalore genei medium range ladder, (Lane 2) Supernatant, (Lane 3) Unbound fraction, (Lane 4) Wash fraction, (Lane 5 and 6) 35 kDa purified AiSQS.

**Accession Numbers**

GeneBank Accession numbers used for construction of phylogenetic analysis are *A.thaliana* AtGDS (NP_001031483), *C. roseus* CrGDS.LSU (AGL91645), *H. lupulus* HlGDS.LSU (ACQ90682), *M. x piperita* MpGDS.LSU (ABW86879), *A. majus* AmGDS.LSU (AAS82860), *C. roseus* (CrGDS, AGL91647), *G. aurea* (GaGDS, EPS58436), *S. lycopersicum* (SlGDS, ABB88703), *S. grosvenorii* (SgGDS, AEM42978), *M. indica* (MiGDS, AFJ52721), *C. sinensis* (CsGDS, CAC16851), *H. brasiliensis* (HbGDS, BAF98299), *T. cacao* (TcGDS, XP_007016031), *G. sulphuraria* (GsGDS, XP_005708177), *A. deanei*, (AdGDS, EPY38369), *S. culicis*, (ScGDS, EPY32675), *L. gelidum*, (LgGDS, AFS41065), *L. albus* (LaFDS, AAA86687), *G. arboretum* (GaFDS, CAA72793), *C. roseus* (CrFDS, ADO95193), *A. thaliana* (AtFDS1, NP_199588), *S. album* (SaFDS, AGV01244), *M. indica* (MiFDS, AFJ52720), *Z. mays* (ZmFDFS, AAQ14871), *T. reesei*, (TrFDS, AFX82678), *R. toruloide*, (RtFDS, EMS21600), *L. chrysorrheus* (LcFDS, BAD15361), *X. laevis* (XlFDS, NP_001090113), *H. sapiens*(HsFDS, P14324), *B. Taurus* (BtFDS, AAL58886), *C. sinensis* (CsFDS, GAA49070) and *E. coli* (EcFDS, BAA00599). *G. max* (GmSQS, NP_001236365), *P. vulgaris* (PvSQS, AHA84150), *L. japonicas* (LjSQS, BAC56854), *E. pekinensis* (EpSQS, AFT92039), *P. tenuifolia* (PtSQS, ABG66304), *C. annuum* (CaSQS, AAD20626), *N. tabacum* (NtSQS, AAB08578) *C. borivilianum* (CbSQS, AFN61199), *H. sapiens* (HsSQS, NP_001274672), *M. musculus*, (MmSQS, NP_034321.2), *R. norvegicus* (RnSQS, NP_062111), *C. glabrata* (CgSQS, BAB12207), *S. cerevisiae*, (ScSQS, AAA34597), *A. gossypii*, (AgSQS, AAS53815), *Y. lipolytica*, (YlSQS, AAD22408), *N. crassa*, (NcSQS, ESA41923), *F. fujikuroi* (FfSQS, ABX64425) and *U. maydis* (UmSQS, CAA68054).

Table S1. Predicted genes for Triterpenoid back bone biosynthesis.

| **Predicted Genes for Triterpenoid Backbone Biosynthesis** | | **Blastx Results** |
| --- | --- | --- |
| **Mevalonate Pathway** | |  |
| Acetyl-CoA C-acetyltransferase [EC:2.3.1.9] | Neem_transcript_6172 | 96% Similarity with acetyl-CoA C-acetyltransferase *Hevea brasiliensis* [BAF98276.1] |
|  | Neem_transcript_14672 | 90% Similarity with acetyl Co-A acetyltransferase *Hevea brasiliensis* [AAL18924.1] |
| Hydroxymethylglutaryl-CoA synthase [EC:2.3.3.10] | Neem_transcript_13206 | 93% Similarity with hydroxy methylglutaryl-CoA synthase *Hevea brasiliensis* [BAF98279.1] |
| Hydroxymethylglutaryl-CoA reductase (NADPH) [EC:1.1.1.34] | Neem_transcript_11884 | 91% Similarity with 3-hydroxy-3-methylglutaryl coenzyme A reductase 1 *Dimocarpus longan* [AET72044.1] |
|  | Neem_transcript_21736 | 91% Similarity with 3-hydroxy-3-methylglutaryl coenzyme A reductase 2 *Dimocarpus longan* [AET72045.1] |
| Mevalonate kinase [EC:2.7.1.36] | Neem_transcript_9934 | 90% Similarity with mevalonate kinase *Hevea brasiliensis* |
| Phospho mevalonate kinase [EC:2.7.4.2] | Neem_transcript_27403 | 88% Similarity with PREDICTED: phospho mevalonate kinase *Vitis vinifera* [XP_002275808.1] |
| Diphosphomevalonate decarboxylase [EC:4.1.1.33] | Neem_transcript_5109 | 93% Similarity with diphosphomevelonate decarboxylase *Hevea brasiliensis* [BAF98285.1] |
| Isopentenyl-diphosphate delta-isomerase [EC:5.3.3.2] | Neem_transcript_31626 | 96% Similarity with isopentenyl diphosphate isomerase *Bupleurum chinense* [ACV74320.1] |
| **Non Mevalonate Pathway (MEP/DOXP pathway)** | |  |
| 1-deoxy-D-xylulose-5-phosphate synthase [EC:2.2.1.7] | Neem_transcript_584 | 94% Similarity with 1-deoxyxylulose-5-phosphate synthase, putative *Ricinus communis* [XP_002516843.1] |
|  | Neem_transcript_13351 | 96% Similarity with 1-deoxyxylulose-5-phosphate synthase, putative *Ricinus communis* [XP_002514364.1] |
|  | Neem_transcript_23240 | 92% Similarity with 1-deoxyxylulose-5-phosphate synthase, putative *Ricinus communis* [XP_002532384.1] |
| 1-deoxy-D-xylulose-5-phosphate reductoisomerase [EC:1.1.1.267] | Neem_transcript_31593 | 94% Similarity with 1-deoxy-D-xylulose 5-phosphate reductoisomerase, chloroplast precursor, putative *Ricinus communis* [XP_002511399.1] |
| 2-C-methyl-D-erythritol 4-phosphate cytidylyl transferase [EC:2.7.7.60] | Neem_transcript_19227 | 88% Similarity with 2-C-methyl-D-erythritol 4-phosphate cytidylyl transferase *Hevea brasiliensis* [BAF98291.1] |
| 4-diphosphocytidyl-2-C-methyl-D-erythritol kinase [EC:2.7.1.148] | Neem_transcript_4316 | 89% Similarity with 4-diphosphocytidyl-2-C-methyl-d-erythritol kinase, putative *Ricinus communis* [XP_002523216.1] |
| 2-C-methyl-D-erythritol 2,4-cyclodiphosphate synthase [EC:4.6.1.12] | Neem_transcript_24304 | 88% Similarity with 2-C-methyl-D-erythritol 2,4-cyclodiphosphate synthase *Citrus jambhiri* [BAF73931.1] |
| (E)-4-hydroxy-3-methylbut-2-enyl-diphosphate synthase [EC:1.17.7.1] | Neem_transcript_14312 | 95% Similarity with 4-hydroxy-3-methylbut-2-en-1-yl diphosphate synthase *Hevea brasiliensis* [BAF98296.1] |
| 4-hydroxy-3-methylbut-2-enyl diphosphatereductase [EC:1.17.1.2] | Neem_transcript_350 | 95% Similarity with PREDICTED: 4-hydroxy-3-methylbut-2-enyl diphosphatereductase *Vitis vinifera* [XP_002284659.1] |
| **Prenyl Pyrophosphate Synthase** | |  |
| Geranyl diphosphate synthase [EC:2.5.1.1] | Neem_transcript_10912 | 88% Similarity with geranyl diphosphat synthase *Quercus robur* [CAC20852.1] |
|  | Neem_transcript_10001 | 95% Similarity with geranyl diphosphate synthase *Hevea brasiliensis* [BAF98300.1] |
| Farnesyl diphosphate synthase [EC: 2.5.1.10] | Neem_transcript_25722 | 90% Similarity with farnesyl pyrophosphate synthase *Cyclocarya paliurus* [ACY80695.1] |
| Geranylgeranyl diphosphate synthase [EC: 2.5.1.29] | Neem_transcript_1166 | 72% Similarity with geranylgeranyl pyrophosphate synthase 1 *Solanum lycopersicum* [NP_001234087.1] |
|  | Neem_transcript_3894 | 86% Similarity with geranylgeranyl pyrophosphate synthase, putative *Ricinus communis* [XP_002529802.1] |
|  | Neem_transcript_16200 | 93% Similarity with geranylgeranyl diphosphate synthase *Medicago sativa* [ADG01841.1] |
|  | Neem_transcript_16736 | 69% Similarity with geranylgeranyl pyrophosphate synthase, *Jatropha curcas* [ADD82422.1] |
|  | Neem_transcript_28215 | 72% Similarity with geranylgeranyl pyrophosphate synthase *Nicotiana tabacum*  [ADD49735.1] |
|  | Neem_transcript_30369 | 81% similarity with geranylgeranyl pyrophosphate synthase 1 *Solanum pennellii* [ADZ24718.1] |
|  | Neem_transcript_18547 | 79% Similarity with geranylgeranyl pyrophosphate synthase, putative *Ricinus communis* [XP_002531191.1] |
| **Triterpene Related** | |  |
| Farnesyl-diphosphate farnesyl transferase [EC:2.5.1.21] | Neem_transcript_33869 | 79% Similarity with squalene synthase *Glycine max* [NP_001236365.1] |
| Squalene monooxygenase [EC:1.14.13.132] | Neem_transcript_11071 | 91% Similarity with squalene monooxygenase putative *Ricinus communis* [XP_002530610] |
|  | Neem_transcript_18229 | 90% Similarity with PREDICTED: squalene monooxygenase *Vitis vinifera* [XP_002271528] |
|  | Neem_transcript_18980 | 90% Similarity with squalene monooxygenase, putative *Ricinus communis* [XP_002510043] |
| Triterpenecyclases | Neem_transcript_28920 | 86% Similarity with Beta-amyrin synthase *Betula platyphylla* [Q8W3Z1.1] |
|  | Neem_transcript_27436 | 92% Similarity with cycloartenol synthase *Betula platyphylla* [Q8W3Z3] |
| Putative CYP related to triterpenoid biosynthesis | Neem_transcript_26034 | 44% Similarity with Beta-amyrin 11-oxidase *Glycyrrhiza uralensis* [BAG68929.1] |
|  | Neem_transcript_26318 | 65% Similarity with Dammarenediol 12-hydroxylase *Panax ginseng* [AEY75213.1] |
|  | Neem_transcript_34861 | 62% Similarity with Protopanaxadiol 6-hydroxylase *Panax ginseng* [AFO63031.1] |
|  | Neem_transcript_10225 | 78% Similarity with Cytochrome P450 CYP72A219 *Panax ginseng* [AEY75218.1] |
|  | Neem_transcript_38933 | 53% Similarity with *Panax ginseng* [AFO63032.1] |
|  | Neem_transcript_23030 | 59% Similarity with Cytochrome P450 CYP736A12 *Panax ginseng* [AEY75215.1] |

Table S2. Present Identity Matrix of AiGDS with plant Homomeric GDS and Heteromeric GDS Larger subunits

|  |  | 1 | 2 | 3 | 4 | 5 | 6 | 7 | 8 | 9 | 10 |
| --- | --- | --- | --- | --- | --- | --- | --- | --- | --- | --- | --- |
| 1 | AiGDS | 100 |  |  |  |  |  |  |  |  |  |
| 2 | CsGDS | 89.72 | 100 |  |  |  |  |  |  |  |  |
| 3 | MiGDS | 83.57 | 88.79 | 100 |  |  |  |  |  |  |  |
| 4 | CrGDS | 76.74 | 83.18 | 75.78 | 100 |  |  |  |  |  |  |
| 5 | AtGDS | 71.15 | 80.69 | 70.74 | 68.02 | 100 |  |  |  |  |  |
| 6 | TcGDS1 | 71.25 | 80.69 | 70.59 | 70.52 | 65.53 | 100 |  |  |  |  |
| 7 | CrGDS.LSU | 26.22 | 27.86 | 25 | 26.59 | 23.7 | 26.04 | 100 |  |  |  |
| 8 | HlGD.SLUS | 27.81 | 29.39 | 26.25 | 27.76 | 26.57 | 28.27 | 72.48 | 100 |  |  |
| 9 | MpGDS.LSU | 25.87 | 28.63 | 24.93 | 26.1 | 24.63 | 27.03 | 71.39 | 67.57 | 100 |  |
| 10 | AmGDS.LSU | 24.41 | 27.86 | 25.22 | 25.37 | 24.48 | 25.23 | 75 | 69.97 | 70.65 | 100 |

Table S3. Primers and vectors used for cloning of AiGDS, AiFDS and AiSQS and RT-PCR primers of 18S rRNA and AiSQS.

| **Primers and vectors used for cloning** | | |
| --- | --- | --- |
| Gene name | Primers | Vectors |
| AiGDS | Forward primer.  ATGACCGGATCCATGTTATTTTCTCGTG  Reverse primer.  CATGTCGAGCTCCTATTTATTTCTTGTGATG | pET32a |
| AiFDS | Forward primer.  ATGAGCGGATCCATGAGTGATCTGCATTCC  Reverse primer.  ACAGATCTCGAGTTACTTCTGCCTCTTG |  |
| AiSQS | Full length forward primer.  CACCGGGAGTTTGGGAGCGGTT  Full length reverse primer.  GTTGTTTGGTCGGTTGGCTG | pCR Blunt and pRSET B |
|  | Truncated Forward primer.  GCTTCTGTTACTCTATGCTT  Truncated reverse primer.  TTATGGATCATTCTCGTTGATCT | pCR Blunt and pET28c |
| **RT-PCR** | | |
| 18S rRNA | Forward primer.  GCACGCGCGCTACAATGAAAG  Reverse Primer.  GTCTGTACAAAGGGCAGGGACG | |
| GAPDH | Forward primer.  TCGGAATCAACGGTTTTGGAA  Reverse Primer.  CACTTGACCGTGAACACTGT | |
| AiSQS | Forward primer.  TGAGCAGGGTGGAAGCAATA  Reverse primer.  CGGTTGGCTGAGAGGTAAGC | |
| AiGDS (Neem_transcript_10912) | Forward primer.  AGTTCCCTGAGTTGCGTAAAG  Reverse primer.  TCATCGTTGCTTTCTGGTAGAG | |
| AiFDS | Forward primer.  GGTGCATCGAATGGCTTCAA  Reverse primer.  GTGCACATGGTTGCGTAGAA | |
| Neem_transcript_10001 | Forward primer.  GCCATATTAGGAGGTGGAAGTG  Reverse primer.  GTCGAACCTGCCTTTGATTTG | |

Table S4. Buffers used for AiGDS, AiFDS and AiSQS protein purification.

| **Expression and purification** | | | | | |
| --- | --- | --- | --- | --- | --- |
| Gene Name | Expression host and expression condition | Lysis buffer | Wash buffer | Elution buffer | Desalting buffer |
| AiGDS | Lemo21(DE3) cells,  1mM IPTG, 16 ºC for 12hr | 100 mM MOPSO, 400 mM NaCl, 0.5% w/v CHAPS, 10% v/v glycerol, 0.5 mM PMSF, 1 mg/mL lysozyme, pH 7.4, | 100 mM MOPSO, 400 mM NaCl, 100 mM imidazole, 10% v/v glycerol, pH 7.4 | 50 mM MOPSO, 300 mM NaCl, 250 mM imidazole, 10% v/v glycerol, 0.2% w/v CHAPS, pH 7.4 | 50 mM MOPS, 100 mM KCl, 10% v/v glycerol, pH-7.4 |
| AiFDS | BL21(DE) cells,  1mM IPTG, 16 ºC for 12hr | 50 mM NaH_2_PO_4_, 300 mM NaCl, 0.2% w/v CHAPS, 10 mM MgCl_2_, 10% v/v glycerol, pH 7.4, 0.5 mM PMSF and 1 mg/mL lysozyme | 50 mM NaH_2_PO_4_, 300 mM NaCl,  50 mM Imidazole,  10% (V/V) glycerol, pH- 7.4 | 50 mM NaH_2_PO_4_, 300 mM NaCl, 250 mM imidazole, 10% glycerol, pH 7.4 | 25 mM HEPES, 100 mM KCl, 10% v/v glycerol, pH-7.4 |
| AiSQS  Truncated | BL21 (DE3) cells, 1mM IPTG, 16 ºC for 12hr | 50 mM Tris-HCl, 500 mM NaCl, 20 mM imidazole, 1 mM PMSF and 1 mg/mL lysozyme, pH 7.4 | (50 mM Tris-Cl, 500 mM NaCl, 20 mM imidazole, pH 7.4 | 50 mM Tris-HCl, 500 mM NaCl, 500 mM imidazole, pH 7.4 | 50 mM Tris-HCl, 200 mM KCl, 20% glycerol, pH 7.4 |
| AiSQS | BL21 Star (DE3) cells, 0.1mM IPTG, 16 ºC for 12hr | 100 mM NaH_2_PO_4_, 500 mM NaCl, 20 mM imidazole, 1 mM PMSF, 1% w/v CHAPS, 50% v/v glycerol, pH 7.4 and 1 mg/mL lysozyme | 100 mM NaH_2_PO_4_, 500 mM NaCl, 20 mM imidazole, 20% v/v glycerol, 1% CHAPS, pH 7.4 | 100 mM NaH_2_PO_4_, 500 mM NaCl, 500 mM imidazole 1% CHAPS and 20% v/v glycerol, pH 7.4 | 50 mM NaH_2_PO_4_, 200 mM KCl and 20% glycerol, pH 7.4 |

Table S5. TargetP analysis Neem_transcript_10912 (AiGDS) and Neem_Transcript_10001

| **Name** | **Len** | **cTP** | **mTP** | **SP** | **other** | **Loc** | **RC** |
| --- | --- | --- | --- | --- | --- | --- | --- |
| Neem_transcript_10912 | 420 | 0.068 | 0.882 | 0.007 | 0.064 | M | 1 |
| Neem_transcript_10001 | 306 | 0.18 | 0.29 | 0.106 | 0.204 | M | 5 |
| Cutoff |  | 0 | 0 | 0 | 0 |  |  |
